# Effects of Synbiotic Supplementation on Chronic Inflammation and the Gut Microbiota in Obese Patients with Type 2 Diabetes Mellitus: A Randomized Controlled Study

**DOI:** 10.3390/nu13020558

**Published:** 2021-02-08

**Authors:** Akio Kanazawa, Masanori Aida, Yasuto Yoshida, Hideyoshi Kaga, Takehiro Katahira, Luka Suzuki, Shoko Tamaki, Junko Sato, Hiromasa Goto, Kosuke Azuma, Tomoaki Shimizu, Takuya Takahashi, Yuichiro Yamashiro, Hirotaka Watada

**Affiliations:** 1 Department of Metabolism & Endocrinology, Graduate School of Medicine, Juntendo University, Tokyo 113-8421, Japan; hkaga@juntendo.ac.jp (H.K.); tkatahi@juntendo.ac.jp (T.K.); rusuzuki@juntendo.ac.jp (L.S.); tamaki-ktz@umin.ac.jp (S.T.); jsato@juntendo.ac.jp (J.S.); gokky@juntendo.ac.jp (H.G.); kosuke@juntendo.ac.jp (K.A.); tomoaki@juntendo.ac.jp (T.S.); hwatada@juntendo.ac.jp (H.W.); 2 Food Research Department, Yakult Central Institute, Tokyo 186-8650, Japan; masanori-aida@yakult.co.jp (M.A.); yasuto-yoshida@yakult.co.jp (Y.Y.); 3 Yakult Honsha European Research Center for Microbiology, 9052 Gent-Zwijnaarde, Belgium; takuya.takahashi@yher.be; 4 Probiotics Research Laboratory, Graduate School of Medicine, Juntendo University, Tokyo 113-8421, Japan; yamasiro@juntendo.ac.jp; 5 Center for Therapeutic Innovations in Diabetes, Graduate School of Medicine, Juntendo University, Tokyo 113-8421, Japan; 6 Center for Identification of Diabetic Therapeutic Targets, Graduate School of Medicine, Juntendo University, Tokyo 113-8421, Japan; 7 Sportology Center, Graduate School of Medicine, Juntendo University, Tokyo 113-8421, Japan

**Keywords:** synbiotic, probiotic, galacto-oligosccharides (GOSs), chronic inflammation, gut microbiota, type 2 diabetes

## Abstract

The aim of this study was to investigate the effects of 24-week synbiotic supplementation on chronic inflammation and the gut microbiota in obese patients with type 2 diabetes. We randomized 88 obese patients with type 2 diabetes to one of two groups for 24 weeks: control or synbiotic (*Lacticaseibacillus paracasei* strain Shirota (previously *Lactobacillus casei* strain Shirota) and *Bifidobacterium breve* strain Yakult, and galactooligosaccharides). The primary endpoint was the change in interleukin-6 from baseline to 24 weeks. Secondary endpoints were evaluation of the gut microbiota in feces and blood, fecal organic acids, high-sensitivity C-reactive protein, lipopolysaccharide-binding protein, and glycemic control. Synbiotic administration for 24 weeks did not significantly affect changes in interleukin-6 from baseline to 24 weeks (0.35 ± 1.99 vs. −0.24 ± 1.75 pg/mL, respectively). Relative to baseline, however, at 24 weeks after synbiotic administration there were positive changes in the counts of *Bifidobacterium* and total lactobacilli, the relative abundances of *Bifidobacterium* species such as *Bifidobacterium adolescentis* and *Bifidobacterium pseudocatenulatum*, and the concentrations of acetic and butyric acids in feces. No significant changes in inflammatory markers were found in the synbiotic group compared to the control group. However, synbiotic administration at least partially improved the gut environment in obese patients with type 2 diabetes.

## 1. Introduction

Changes in the gut microbiota [1] and its derived metabolites are closely associated with insulin sensitivity [2,3], incretin secretion [4,5], and energy homeostasis [6]. Thus, the gut microbiota has been attracting much attention in metabolic diseases such as obesity and type 2 diabetes mellitus (T2DM).

Previously, we reported the presence of gut dysbiosis and bacterial translocation in patients with T2DM [7]. In a subsequent randomized controlled trial, we found that probiotic administration reduced the translocation of gut bacteria to blood in patients with T2DM [8]. Plasma lipopolysaccharide (LPS) from gram-negative bacteria, like translocation of gut bacteria, is an inflammatory mediator that contributes to insulin resistance [9,10]. Translocated LPS in the systemic circulation binds to LPS-binding protein (LBP), which is a marker for metabolic syndrome [11,12], and our previous study showed clear positive associations between plasma levels of interleukin-6 and LBP in patients with T2DM [13]. Furthermore, we found higher plasma LBP levels in T2DM patients with obesity and poor glycemic control [13]. Therefore, a possible therapeutic approach in obese patients with T2DM is to control low-grade chronic inflammation by reducing not only translocation of gut bacteria but also the levels of endotoxins such as LPS. According to the previous review [14], it has been reported that probiotics administration in patients with metabolic syndrome resulted in improvements in body mass index, lipid, and glucose metabolism, and probiotics also positively affected inflammatory markers such as interleukine-6.

While probiotics may have various beneficial effects on metabolic disease as mentioned above, the combination of one or more probiotics and prebiotics, a mixture generally referred to as a synbiotic, may confer further significant benefits in the human gut environment. Indeed, perioperative synbiotic treatment consisting of two probiotic strains, *Lacticaseibacillus paracasei* (the previous taxonomic nomenclature was *Lactobacillus casei*) strain Shirota (LcS) and *Bifidobacterium breve* strain Yakult (BbrY), along with galacto-oligosaccharides (GOS), significantly prevented postoperative infectious complications due to reduction of bacterial translocation [15]. Thus, our hypothesis is that synbiotics might be expected to effectively inhibit bacterial translocation in metabolic diseases, and subsequently might reduce chronic inflammation. Here, we performed a 24-week, interventional, randomized controlled study to investigate the effects of daily intake of a synbiotic comprising LcS, BbrY, and GOS on chronic inflammation, gut microbiota, fecal organic acids, and bacterial translocation in obese patients with T2DM.

## 2. Materials and Methods

### 2.1. Participants

A total of 88 obese patients with T2DM were recruited from the outpatient clinic of Juntendo University Hospital (*n* = 72) and International Good Will Hospital (*n* = 16) between July 2018 and April 2019. The following inclusion criteria were applied at study registration: (1) age ≥ 30 but <80 years, (2) HbA1c (NGSP) ≥ 6.0 but <9.0%, (3) body mass index (BMI) ≥ 25.0 kg/m^2^, and (4) treatment with only diet and exercise or medicines. The selected patients were excluded from the study if any of the following conditions were diagnosed at registration: (1) serious kidney disease (serum creatinine level ≥ 1.5 mg/dL and/or hemodialysis), (2) serious liver disease excluding fatty liver, (3) inflammatory bowel disease, (4) type 1 diabetes mellitus, (5) past history of digestive surgery, (6) allergy to milk, (7) treatment with an α-glucosidase inhibitor, and (8) unsuitability for the study (i.e., irregular visits). This study was registered in the University Hospital Medical Information Network Clinical Trials Registry, a non-profit organization in Japan, and it meets the requirements of the International Committee of Medical Journal Editors (UMIN000032057, registration date: 2 April 2018).

### 2.2. Study Design

The primary endpoint was the change in the level of interleukin-6 (IL-6) from baseline to the end of the study. The secondary endpoints were changes from baseline to the end of the study in high-sensitivity C-reactive protein (hs-CRP), LBP, bacterial counts in feces and blood, fecal organic acid concentrations, diversity of the gut microbiota in feces, and the levels of fasting blood glucose, HbA1c, and lipids. Our previous study showed that the IL-6 level was 1.8 ± 0.9 pg/mL before probiotic administration and 0.20 ± 1.00 pg/mL after the intervention [8]. In another study, the plasma IL-6 level was reduced by around 40% after synbiotic intervention [15]. Based on these previous reports, we assumed that the effect size could be 0.65 ± 1.00 pg/mL between control and synbiotic groups. With a two-sided α level of 5% and power (1-β) of 80%, at least 78 patients (39 patients in each group) would permit the detection of IL-6 changes. Therefore, after considering the possibility of a 10% dropout rate, we recruited 88 patients who were then randomly assigned to either a control group or synbiotic group for 24 weeks. Randomization was performed using a dynamic allocation method based on HbA1c and BMI at baseline (Soiken, Inc., Osaka, Japan). During the study period, physicians in charge were permitted to change patients’ diabetes medications as needed. Samples for biochemical assays and for analysis of the gut microbiota in blood and feces and of fecal organic acids were obtained after overnight fasts at each hospital visit (0, 12, and 24 weeks). At 24 weeks, patients with synbiotic intake rate <60% and those who took antibiotics within 1 week before the collection of the fecal samples were excluded from the per protocol set analysis.

### 2.3. Synbiotic Supplementation

As synbiotic intervention, the following agents were administered orally: 3.0 g dry powder containing at least 3 × 10^8^ living *Lacticaseibacillus paracasei* YIT 9029 (strain Shirota: LcS) organisms, 3 × 10^8^ living *Bifidobacterium breve* YIT 12272 (BbrY) organisms, and 7.5 g GOS per day (product name: Yakult Super Synbiotics LBG-P, Yakult Honsha Co., Ltd., Tokyo, Japan). Patients were instructed to take the synbiotic twice a day (2.0 g dry powder and 5.0 g GOS at breakfast and 1.0 g dry powder and 2.5 g GOS at dinner). The nutritional composition of 3.0 g dry powder and of 7.5 g GOS was as follows: energy, 12.0 kcal and 54.9 kcal; protein, 0.03 g and 0.00 g; lipids, 0.0 g and 0.0 g; and carbohydrates, 3.0 g and 15.3 g, respectively. The participants in the synbiotic group consumed the aforementioned dose every day for 24 weeks; this was verified by up to three telephone calls to each patient, as necessary, just before their hospital visits (0, 12, and 24 weeks). In addition, each patient in the synbiotic group was instructed to keep a diary of synbiotic intake, and the control group was told not to take any synbiotic. During the study period, all participants were prohibited from consuming any other probiotics or prebiotics. In addition, the participants in the synbiotic group were instructed to reduce their calorie intake by about 60 kcal/day considering the additional calories from the synbiotic agent.

### 2.4. Determination of Bacterial Count by rRNA-Targeted Reverse Transcription-Quantitative PCR (RT-qPCR) and qPCR

We examined the gut microbiota composition and plasma levels of the gut bacteria using Yakult Intestinal Flora-SCAN (YIF-SCAN^®^), a bacterial rRNA-targeted RT-qPCR system [16,17,18]. The threshold cycle values in the linear range of the assay were applied to the standard curve to obtain the corresponding bacterial cell count in each nucleic acid sample. These data were then used to determine the number of bacteria per sample. The specificity of the RT-qPCR assay using group-, genus- or species-specific primers was determined as previously described [16,17,18,19]. For the enumeration of LcS and BbrY in feces, qPCR analysis was performed using previously described methods [20,21]. The sequences of the primers are listed in Table S1.

### 2.5. 16S rRNA Gene Sequencing for Microbiota Analysis

Bacterial DNA in feces was extracted as previously described [22]. The V1-2 regions of the 16S rRNA gene in each sample were amplified using the forward 27Fmod2 and reverse 338R primers [23]. Amplification and sequencing were performed using an ABI PRISM^®^ 7500 Real-Time PCR System (Applied Biosystems, Framingham, MA, USA) and a MiSeq sequencer with MiSeq Reagent Kits v2 (Illumina, SanDiego, CA, USA) as previously described [24]. The sequences generated from the MiSeq platform were analyzed using the open-source software package Quantitative Insights Into Microbial Ecology 2 (QIIME2) (2020.2) [25], and the SILVA 138 database (https://www.arb-silva.de/) was used to annotate taxonomic information. α-diversities represented as the number of observed operational taxonomic units (OTUs), the Shannon index, and phylogenetic diversity (PD) were estimated for 5000 randomly selected sequences to account for differences in sampling effort between the samples.

### 2.6. Measurement of Organic Acids and pH in Fecal Samples

The pH of stool specimens was analyzed using a handheld pH meter (model IQ150; IQ Scientific Instruments, San Diego, CA, USA). The concentration of organic acids in each sample was measured using a high-performance liquid chromatography system equipped with 432 electroconductivity detectors (Waters), as previously described [19]. In addition, all assays were performed blindly, including those involving the gut microbiota and organic acids.

### 2.7. Biochemical Assays

Serum lipids (total cholesterol, high-density lipoprotein cholesterol, and triglycerides), fasting blood glucose, and HbA1c were measured with standard techniques. The plasma levels of hs-CRP and IL-6 were measured by latex nephelometry, chemiluminescent enzyme immunoassay, and enzyme-linked immunosorbent assay, respectively, at a private laboratory (SRL Laboratory, Tokyo, Japan). The plasma level of LBP was measured using a Human LBP ELISA Kit (RayBiotech, GA, USA).

### 2.8. Statistical Analyses

All statistical analyses were performed by a private company (Soiken, Inc., Osaka, Japan) with SAS software version 9.4 (SAS Institute, Cary, NC, USA). Normally distributed data were expressed as mean ± standard deviation and were analyzed by the t-test. Data with skewed distribution were expressed as median (interquartile range) and were analyzed by the Wilcoxon rank sum test. The detection rates of fecal and blood bacteria and fecal organic acids in both groups were analyzed by Fisher’s exact probability test. *P* < 0.05 was considered statistically significant. For microbiota analysis, RT-qPCR-negative samples were analyzed using half values of the lower limit (logarithm) that each corresponding primer sets could detect. Then, for enumeration of LcS and BbrY in feces, qPCR-negative samples were excluded from the statistical analysis. Differences in the relative abundance of microbial features were determined by linear discriminant analysis (LDA) effect size (LEfSe) analysis using the Galaxy web application (http://huttenhower.sph.harvard.edu/galaxy/) [26]. Bacterial abundance profiles were calculated at taxonomic levels from phylum to species in terms of percent abundance, and a logarithmic LDA score  ≥  2.0 was used as a threshold.

## 3. Results

### 3.1. Baseline Characteristics

**Figure 1:**
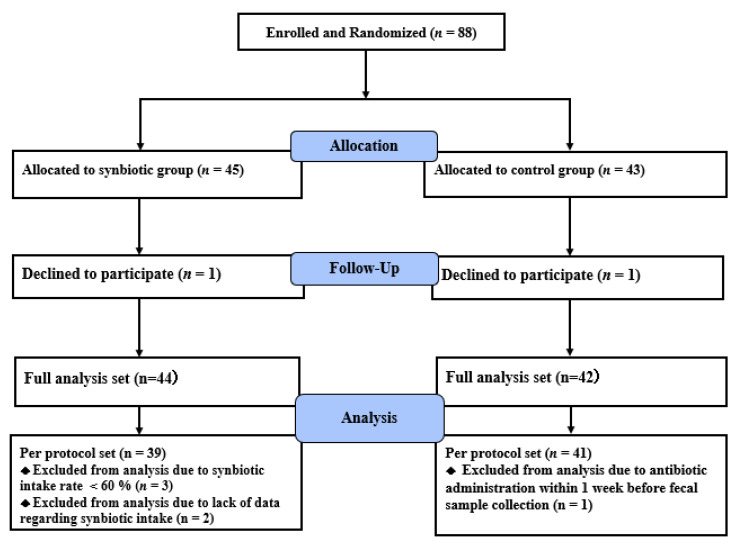
Flow diagram of patient recruitment. In total, 88 patients were randomly allocated to either the synbiotic group or control group. One patient each in the synbiotic and control groups declined to participate. The remaining 86 patients were followed up for 24 weeks.

Figure 1 shows the study flow diagram. Of the 88 patients recruited in this study, 45 were assigned to the synbiotic group and 43 to the control group. One patient in the synbiotic group declined participation after randomization, and therefore 44 patients completed the 24-week intervention. In the control group, one patient declined participation and thus 42 patients completed the 24-week trial. The baseline characteristics of the patients who completed the study are summarized in Table 1. The mean age in the synbiotic group was significantly higher than that in the control group, while the other parameters were comparable between the two groups. Five patients were excluded from the per protocol set analysis in the synbiotic group due to low or unknown compliance with synbiotic intake, and one was excluded in the control group due to antibiotic treatment before the collection of fecal samples.

### 3.2. Serial Changes in Inflammatory Markers, Glycemic Control, and Lipid Levels

As shown in Table 2, the two groups demonstrated no significant changes in IL-6, LBP, or hs-CRP from baseline to 24 weeks. Regarding glycemic control, the synbiotic group showed significantly higher levels of fasting blood glucose and HbA1c at 12 weeks compared with the control group, and also a significant positive change in HbA1c from baseline to 12 weeks. However, glycemic control at 24 weeks did not differ between the two groups. In addition, BMI and lipid levels did not change significantly during the study period in either group. Finally, the primary outcome, namely the change in IL-6 level from baseline to 24 weeks, did not differ significantly between the two groups in the per protocol set analysis.

### 3.3. Serial Changes in and Detection Rates of Fecal Microbiota by RT-qPCR and qPCR

Table 3 shows the serial changes in fecal microbiota determined by RT-qPCR. At baseline, the counts of *Bifidobacterium*, *Lactobacillus* (formerly *Lactobacillus gasseri* subgroup), and *Streptococcus* were significantly higher in the synbiotic group compared with the control group, and no other bacteria showed significant differences between the two groups. At 12 weeks, the counts of *Bifidobacterium*, total lactobacilli, and the *Lactobacillus*, *Lacticaseibacillus* (formerly *Lactobacillus casei* subgroup), *Lactiplantibacillus* (formerly *Lactobacillus plantarum* subgroup), and *Limosilactobacillus* (except *L. fermentum*) (formerly *Lactobacillus reuteri* subgroup) were significantly higher in the synbiotic group compared with the control group. Relative to the control group, the synbiotic group also showed significant positive changes from baseline to 12 weeks in the counts of *Bifidobacterium,* total lactobacilli, and the *Lacticaseibacillus* and *Limosilactobacillus*(except *L. fermentum*). At 24 weeks, the counts of total bacteria, *Bifidobacterium*, *Atopobium* cluster, total lactobacilli, and the *Lactobacillus*, *Lacticaseibacillus*, and *Limosilactobacillus* (except *L. fermentum*) were significantly higher in the synbiotic group compared with the control group. Further, relative to the control group, the synbiotic group showed significant positive changes from baseline to 24 weeks in the counts of *Bifidobacterium*, *Prevotella*, total lactobacilli, *Lactobacillus* and *Lacticaseibacillus*, and a significant negative change in *Akkermansia muciniphila*.

Table S2 shows the serial changes in LcS and BbrY determined by qPCR. Although there were no significant differences between the two groups at baseline, the counts and detection rates of these bacteria were significantly higher in the synbiotic group compared with the control group at 12 and 24 weeks.

### 3.4. Serial Changes in Fecal Microbiota by 16S rRNA Gene Sequencing

The relative abundance of each bacterial phylum and the changes from baseline are summarized in Table 4. At 12 weeks, the percentage of Actinobacteriota was significantly higher and that of Bacteroidota was significantly lower in the synbiotic group than in the control group, and the synbiotic group showed a significant positive change from baseline to 12 weeks in the percentage of Actinobacteriota. At 24 weeks, the synbiotic group demonstrated a significantly higher percentage of Actinobacteriota than the control group, and this phylum showed a significant positive change from baseline compared with the control group. Conversely, at 24 weeks, the synbiotic group showed significantly lower percentages of Bacteroidota and Fusobacteriota and significant negative changes from baseline for these phyla and Proteobacteria when compared with the control group.

Microbial diversities represented by phylogenic diversity, observed OTUs, and the Shannon index were transiently decreased at 12 weeks after synbiotic administration; however, these indices did not differ between the two groups at 24 weeks (Table 5).

Serial changes in the relative abundances of 33 bacterial families and 37 bacterial species assigned based on the SILVA database are summarized in Table 6 and Table S3, respectively. The main findings were that the change of Bifidobacteriaceae at 12 and 24 weeks and that of Veillonellaceae at 24 weeks were significantly increased relative to baseline in the synbiotic group compared with the control group. In contrast, the changes of Bacteroidaceae at 12 and 24 weeks and those of Marinifilaceae at 12 weeks and Fusobacteriaceae and Monoglobaceae at 24 weeks were significantly decreased in the synbiotic group compared with the control group (Table 6).

Relative to the control group, the synbiotic group demonstrated significant positive changes in the changes of *Bifidobacterium adolescentis* and *Bifidobacterium pseudocatenulatum* from baseline to both 12 and 24 weeks, as well as of *Veillonella ratti* from baseline to 12 weeks and of *Bacteroides coprocola* and *Megasphaera elsdenii* from baseline to 24 weeks. Furthermore, the synbiotic group showed significant negative changes compared to the control group in the relative abundances of *Bacteroides caccae*, *Roseburia inulinivorans*, and *Phascolarctobacterium faecium* from baseline to 12 weeks, and of *Fusobacterium mortiferum* and *Bacteroides vulgatus* from baseline to 24 weeks (Table S3).

**Figure 2:**
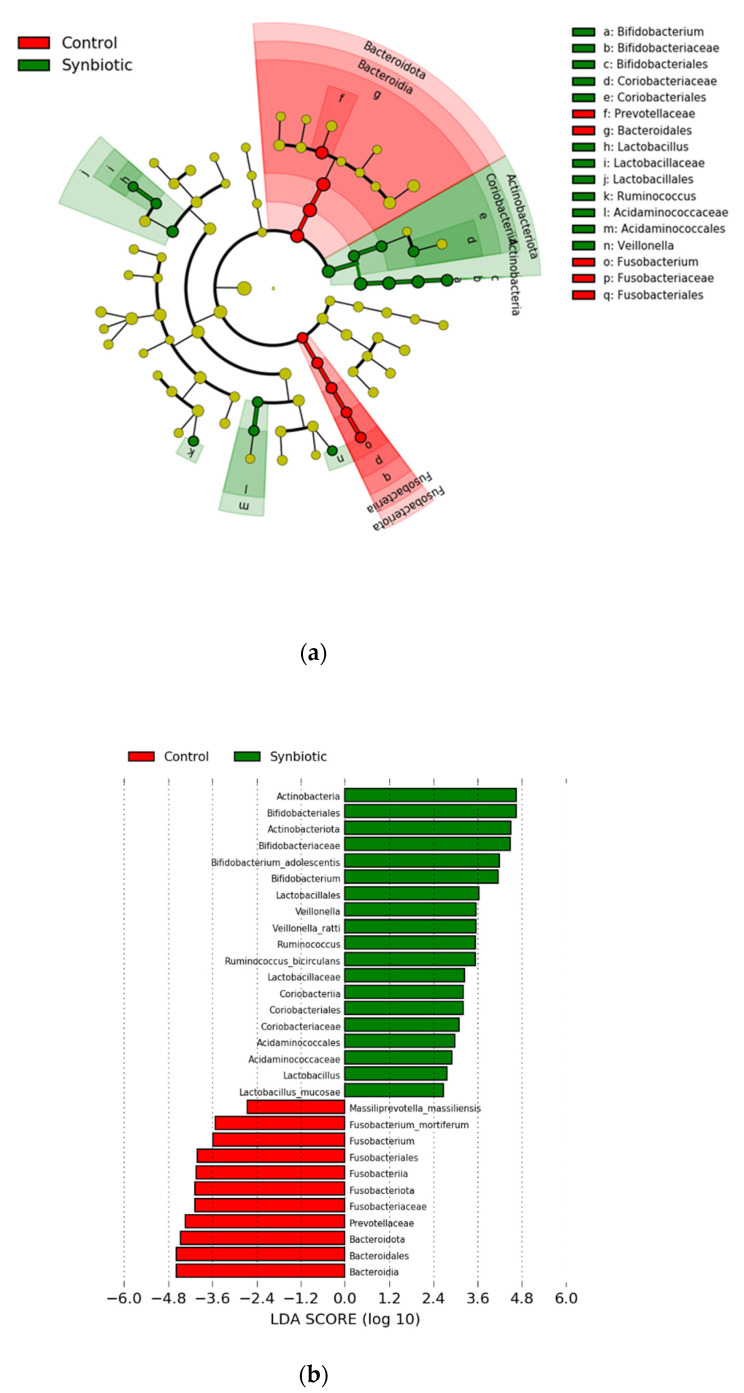
Linear discriminant analysis (LDA) effect size (LEfSe) effect size was used to calculate the taxa that best discriminated between the synbiotic and control groups. (**a**) Expressed in a cladogram, taxa that reached a linear discriminant analysis score (log10) >2.0 are highlighted and labelled accordingly. (**b**) LDA score >2.0 at taxonomic levels from phylum to species.

We applied LEfSe analysis to explore the taxa that best discriminated bacterial populations between the two groups. In the synbiotic group at 24 weeks, we found a pronounced deposition of the Actinobacteriota phylum and Bifidobacteriaceae family, and a high LDA score of this phylum and family, respectively (Figure 2).

### 3.5. Serial Changes in Fecal Organic Acids and pH

The serial changes in fecal organic acids and pH are presented in Table 7. At baseline and 12 weeks, the concentration of propionic acid was significantly lower in the synbiotic group compared with the control group. The concentrations of total organic acids, acetic acids, and butyric acids were significantly increased in the synbiotic group compared to the control group at 24 weeks. Furthermore, relative to the control group, the synbiotic group showed a significant positive change in the concentration of lactic acid at 12 weeks compared to the control group, and the measured concentrations of other organic acids and fecal pH at each visit and their changes from baseline were comparable between the two groups.

### 3.6. Detection Rates of Gut Bacteria in the Blood before and after Synbiotic Administration

Enterobacteriaceae was detected in one patient at 12 and 24 weeks in the control group (detection rate, 2.4%). *Streptococcus* was detected in one patient at 12 weeks in the control group (detection rate, 2.4%), and in one patient at 0, 12, and 24 weeks in the synbiotic group (detection rate, 2.3%). These detection rates were comparable between the two groups.

### 3.7. Adverse Events and Changes in Diabetes Treatment

As summarized by Table S4, adverse events involving the gastrointestinal tract affected four patients in the synbiotic group but none in the control group. In the synbiotic group, two patients underwent new administration, and insulin dose was increased in another patient. In the control group, three patients underwent new administration, and some medications were discontinued or titrated. No patients in either group newly received an α-glucosidase inhibitor, metformin, or thiazolidine, or underwent dose titration of metformin.

## 4. Discussion

The present study assessed the effect of synbiotic administration on several inflammatory markers, including IL-6, LBP, and hs-CRP, in obese patients with T2DM. The results showed no differences in the levels of inflammatory markers between the synbiotic and control groups. So far, several studies have investigated the effects of probiotics/synbiotics on inflammatory markers in T2DM. While some found that IL-6 levels were reduced [27,28], others did not [29,30]. Thus, this issue remains controversial. The reasons for these inconsistencies remain unknown, but they may be due to study differences in probiotics/synbiotics as well as patient age, ethnicity, and eating habits.

In the present study, the effects of a synbiotic on the gut microbiota was investigated quantitatively and qualitatively using RT-qPCR and 16S rRNA amplicon analysis. The relative abundances of Bifidobacteriaceae was significantly increased in feces after synbiotic administration. Of the increased Bifidobacteriaceae, the two species of *Bifidobacterium adolescentis* and *B. pseudocatenulatum* were increased after synbiotic administration. Interestingly, metformin was reported to directly cause the growth of *B. adolescentis* and also *A. muciniphila* [31]. Furthermore, *B. adolescentis* is positively associated with GLP-1 secretion [32] and exhibits inhibitory activity against dipeptidyl peptidase-4 [33]. Therefore, this bacterium might exert incretin-mediated and/or unknown antidiabetic effects via metformin. In addition, some strains of *B. pseudocatenulatum* have beneficial effects on inflammation [34] and metabolism [35]. Taken together, the bacteria that showed an increase in relative abundance in response to synbiotic administration might play important roles in glucose metabolism.

In our previous research using only LcS as a probiotic [8], no changes in fecal organic acids were found in T2DM, but in the present study, which instead used a synbiotic, the fecal acetic acid concentration was significantly increased. It was reported that endogenous *Bifidobacterium* belonging to the predominant obligate anaerobes in humans can grow using the GOS in synbiotics and efficiently produce acetic acids as well as BbrY [36,37]. Therefore, it is considered that GOS might be a key regulator in increasing fecal acetic acids, with BbrY playing a secondary role. It was also demonstrated that short chain fatty acids, especially acetic acid, can improve the function of the gut barrier [38]. Furthermore, Kimura et al. reported that acetic acid promoted glucose metabolism via the activation of G protein-coupled receptor 43, which suppresses insulin signaling in adipocytes [39]. Therefore, increasing the production of acetic acid might also play an important role in glycemic control.

The counts of two lactobacilli genera, specifically the *Lactobacillus* and *Lacticaseibacillus*, also increased after synbiotic administration. This was not surprising for *Lacticaseibacillus*, since it was administered as the LcS probiotic, but that was not the case for *Lactobacillus*. *L. gasseri* is a microorganism that is vaginally transmitted from mother to infant at birth [40], and is considered one of the primary microbiota to be involved in GOS fermentation [41]. In this study, therefore, *L. gasseri* may have utilized the GOS in the synbiotic, leading to an increase in fecal count of *Lactobacillus*. It has been reported that this bacterium has anti-pathogenic activity, for instance via the production of bacteriocin, and that it contributes to the maintenance of gut homeostasis [42]. In addition, a previous study showed a positive correlation between HbA1c and the bacterial count of *Lactobacillus* [43], suggesting important roles of this subgroup in glycemic control. However, the precise mechanism remains unknown, and further studies to investigate the pathophysiological roles of these bacteria in T2DM are necessary.

*B. vulgatus*, which in this study demonstrated a decrease in bacterial count in response to synbiotic administration, was recently identified as the main species driving the association between biosynthesis of branched-chain amino acids and insulin resistance in obese patients [2]. Therefore, a decrease in this bacterium following exposure to a synbiotic might play an important role in insulin resistance in obese patients with T2DM. Although this study suggested that glycemic control might be affected by changes in various gut bacteria, it did not improve after synbiotic administration. One reason may be that changes in glycemic control were difficult to evaluate because the mean HbA1c level at baseline was not very high (synbiotic group, HbA1c 7.4 ± 0.7%).

Interestingly, Bacteroidaceae abundance and *A. muciniphila* count were decreased in this study. These two bacteria are known as mucolytic bacteria [44]. Therefore, it was suggested that endogenous mucolytic Bacteroidaceae and *A. muciniphila* were relatively reduced due to the increase of mucolytic *Bifidobacterium* by synbiotic administration.

Our study has several limitations. First, since the study did not use a double-blind design, patients in the synbiotic group may have been aware of the effects of the synbiotic on the gut microbiota, which might have biased the results. Second, the study did not directly evaluate gut barrier function or plasma LPS levels. Therefore, it remains unknown whether synbiotic administration definitely reduced plasma LPS levels in T2DM. Third, the detection rate (2.4%) of gut bacteria in blood was very low compared with a previous study (22.0%) [13]. Therefore, we could not evaluate the effects of the synbiotic on the translocation of live gut bacteria to the blood. Additionally, it is known that probiotic bacteria regulate intestinal permeability by tumor necrosis factor (TNF)-α-dependent mechanisms [45]. However, in this study plasma levels of TNF-α were not evaluated. Therefore, the evaluation will be necessary for future study.

## 5. Conclusions

In conclusion, 24-week administration of a synbiotic consisting of LcS, BbrY, and GOS did not affect inflammatory markers, but it did at least partially improve the gut environment by increasing the counts of *Bifidobacterium* and *Lactobacillus* and the concentrations of fecal organic acids in obese patients with T2DM.

## Figures and Tables

**Table 1:** Subject characteristics at baseline.

	Control (*n* = 42)	Synbiotic (*n* = 44)
Sex (male/female)	34/8	31/13
Age (years)	55.9 ± 10.7	61.1 ± 11.0 *
BMI (kg/m^2^)	29.1 ± 3.7	29.5 ± 4.4
Systolic blood pressure	130.2 ± 14.9	129.0 ± 14.0
Diastolic blood pressure	79.1 ± 12.1	75.3 ± 9.1
HbA1c (%)	7.3 ± 0.8	7.4 ± 0.7
Fasting blood glucose (mg/dL)	131.7 ± 21.5	140.5 ± 33.6
Fasting C-peptide (ng/mL)	2.50 ± 1.49	2.30 ± 1.18
T-CHO (mg/dL)	196.7 ± 47.5	188.7 ± 34.0
HDL-C (mg/dL)	49.4 ± 10.3	50.8 ± 9.7
TG (mg/dL)	229.0 ± 385.7	141.6 ± 71.3
IL-6 (pg/mL)	2.33 ± 1.27	2.68 ± 2.06
LBP (μg/mL)	5.6 ± 2.5	6.4 ± 4.2
hs-CRP (mg/dL)	1050.0 [450.0, 1800.0]	603.5 [373.5, 1890.0]
Medication for diabetes		
No medication	6 (14.2)	3 (6.8)
Insulin only or with oral therapy	15 (35.7)	13 (29.5)
Oral therapy only		
SU	4 (9.5)	4 (9.1)
Metformin	26 (61.9)	29 (65.9)
Thiazolidine	5 (11.9)	7(15.9)
DPP-4 inhibitor	21(50.0)	21(47.7)
Glinide	4 (9.5)	2 (4.5)
SGLT2 inhibitor	19 (45.2)	23 (52.3)
GLP-1 receptor agonist	5 (11.9)	5 (11.4)

Data are mean ± SD or median [interquartile range: 25%, 75%]. Numbers in parentheses show percentages (%). BMI, body mass index; T-CHO, total cholesterol; HDL-C, high-density lipoprotein cholesterol; TG, triglycerides; hs-CRP, high-sensitivity C-reactive protein; IL-6, interleukin-6; LBP, lipopolysaccharide binding protein; SU, sulfonylurea; DPP-4 inhibitor, dipeptidyl peptidase-4 inhibitor; SGLT2 inhibitor, sodium-dependent glucose cotransporter-2 inhibitor; GLP-1 receptor agonist, glucagon-like peptide-1-receptor agonist. * *p* < 0.05 vs. Control.

**Table 2:** Serial changes in clinical parameters in the control and synbiotic groups.

		Measured Values		Changes	
		12 Weeks	24 Weeks	12 Weeks	24 Weeks
IL-6	Control	2.4 ± 1.2	2.7 ± 2.1	0.1 ± 1.4	0.4 ± 2.0
(pg/mL)	Synbiotic	2.6 ± 1.9	2.5 ± 1.2	0.0 ± 2.1	-0.2 ± 1.8
hs-CRP	Control	914.5 [438.0, 1900.0]	819.5 [304.0, 2300.0]	−12.5 [−425.0, 148.0]	−3.5 [−442.0, 430.0]
(mg/dL)	Synbiotic	729.0 [433.5, 1775.0]	743.5 [341.0, 1820.0]	−26.5 [−398.5, 125.0]	40.0 [−197.0, 327.5]
LBP	Control	7.4 ± 2.5	8.9 ± 4.8	1.8 ± 2.9	3.4 ± 4.2
(μg/mL)	Synbiotic	7.4 ± 3.1	8.3 ± 2.9	1.1 ± 4.5	2.0 ± 4.2
BMI	Control	29.2 ± 3.9	29.4 ± 4.2	0.2 ± 0.6	0.2 ± 0.7
(kg/m^2^)	Synbiotic	29.4 ± 4.6	29.5 ± 4.5	0.0 ± 0.7	0.0 ± 0.7
HbA1c	Control	7.3 ± 0.7	7.4 ± 0.8	0.0 ± 0.5	0.1 ± 0.5
(%)	Synbiotic	7.7 ± 1.0 *	7.6 ± 1.0	0.3 ± 0.7 *	0.2 ± 0.8
Fasting blood glucose	Control	131.2 ± 24.6	135.2 ± 29.9	−0.1 ± 23.5	2.6 ± 26.7
(mg/dL)	Synbiotic	147.5 ± 37.1 *	146.7 ± 41.1	7.0 ± 35.7	6.2 ± 40.4
Fasting C-peptide	Control	2.5 ± 1.5	2.6 ± 1.4	0.0 ± 0.8	0.1 ± 1.2
(ng/mL)	Synbiotic	2.3 ± 1.2	2.4 ± 1.5	0.0 ± 0.9	0.1 ± 0.9
T-CHO	Control	198.4 ± 47.4	194.7 ± 45.5	3.1 ± 24.3	−0.8 ± 37.8
(mg/dL)	Synbiotic	185.1 ± 30.3	189.5 ± 31.8	−3.6 ± 26.2	0.8 ± 25.7
HDL-C	Control	50.4 ± 9.2	49.7 ± 9.2	1.0 ± 6.3	0.2 ± 7.2
(mg/dL)	Synbiotic	50.3 ± 9.5	49.9 ± 9.5	−0.4 ± 5.5	−0.9 ± 5.5
TG	Control	231.2 ± 387.1	211.6 ± 266.6	−1.1 ± 65.5	−10.0 ± 297.7
(mg/dL)	Synbiotic	157.8 ± 115.8	181.6 ± 132.5	16.2 ± 77.6	40.0 ± 94.7

See Table 1 for abbreviations. Data are mean ± SD or median [interquartile range: 25%, 75%]. Each change is expressed as the value measured at 12 and 24 weeks minus the baseline value (0 weeks). * *p* < 0.05 vs. Control.

**Table 3:** Fecal counts of gut microbiota and their changes as determined by RT-qPCR.

		Fecal Bacterial Counts (log_10_ Cells/g)						Changes	
		0 Weeks		12 Weeks		24 Weeks		12 Weeks	24 Weeks
Total bacteria	Control	10.0 ± 0.7	(100.0)	10.0 ± 0.6	(100.0)	9.9 ± 0.5	(100.0)	−0.1 ± 0.6	−0.1 ± 0.7
	Synbiotic	10.1 ± 0.5	(100.0)	10.1 ± 0.6	(100.0)	10.2 ± 0.6 *	(100.0)	0.0 ± 0.7	0.1 ± 0.7
*Clostridium coccoides* group	Control	9.3 ± 0.8	(100.0)	9.2 ± 0.7	(100.0)	8.8 ± 1.7	(95.2)	−0.2 ± 0.7	−0.6 ± 1.6
	Synbiotic	9.3 ± 0.7	(100.0)	9.2 ± 0.8	(100.0)	9.2 ± 0.7	(100.0)	−0.2 ± 1.0	−0.1 ± 0.9
*C. leptum* subgroup	Control	9.1 ± 1.0	(100.0)	9.1 ± 0.8	(100.0)	9.1 ± 0.7	(100.0)	0.0 ± 0.7	0.0 ± 0.9
	Synbiotic	9.2 ± 0.7	(100.0)	9.2 ± 1.2	(97.7)	9.3 ± 0.9	(100.0)	−0.1 ± 1.1	0.1 ± 0.9
*Bacteroides fragilis* group	Control	8.5 ± 1.1	(100.0)	8.2 ± 0.9	(100.0)	8.1 ± 0.9	(100.0)	−0.3 ± 0.9	−0.4 ± 1.0
	Synbiotic	8.5 ± 1.0	(100.0)	8.2 ± 1.2	(97.7)	8.1 ± 0.7	(100.0)	−0.4 ± 1.4	−0.3 ± 1.2
*Bifidobacterium*	Control	8.5 ± 0.9	(100.0)	7.9 ± 1.7	(95.2)	7.9 ± 1.1	(100.0)	−0.5 ± 1.3	−0.6 ± 1.0
	Synbiotic	9.0 ± 0.9 **	(100.0)	9.3 ± 0.8 **	(100.0)	9.4 ± 0.8 **	(100.0)	0.3 ± 0.9 **	0.5 ± 1.1 **
*Atopobium* cluster	Control	8.9 ± 1.2	(97.6)	8.9 ± 0.7	(100.0)	9.0 ± 0.7	(100.0)	0.0 ± 1.2	0.1 ± 10
	Synbiotic	9.2 ± 0.7	(100.0)	9.2 ± 0.8	(100.0)	9.4 ± 0.7 **	(100.0)	0.0 ± 0.7	0.2 ± 0.8
*Prevotella*	Control	6.7 ± 3.1	(61.9)	6.5 ± 3.1	(59.5)	6.5 ± 3.0	(59.5)	−0.2 ± 1.5	−0.2 ± 1.6
	Synbiotic	5.5 ± 2.9	(45.5)	5.9 ± 3.0	(52.3)	5.9 ± 2.8	(54.8)	0.4 ± 1.8	0.6 ± 2.0 *
*C. perfringens*	Control	2.7 ± 1.9	(33.3)	2.3 ± 1.6	(26.2)	2.8 ± 1.8	(38.1)	−0.3 ± 1.7	0.1 ± 2.5
	Synbiotic	2.6 ± 1.8	(34.1)	2.7 ± 1.8	(34.1)	2.6 ± 1.9	(28.6)	0.0 ± 1.7	−0.1 ± 1.9
*Akkermansia muciniphila*	Control	5.9 ± 2.1	(78.6)	5.9 ± 2.0	(83.3)	6.0 ± 2.5	(73.8)	0.1 ± 1.8	0.1 ± 2.1
	Synbiotic	6.3 ± 2.0	(86.4)	5.7 ± 2.0	(77.3)	5.5 ± 2.4	(66.7)	−0.6 ± 2.0	−0.8 ± 2.2 *
Total lactobacilli	Control	5.6 ± 1.7	(95.2)	5.6 ± 1.7	(95.2)	5.7 ± 2.1	(88.1)	0.1 ± 1.1	0.1 ± 1.7
	Synbiotic	6.4 ± 2.0	(95.5)	7.6 ± 1.0 **	(100.0)	7.7 ± 1.0 **	(100.0)	1.3 ± 1.7 **	1.3 ± 1.7 **
*Lactobacillus*	Control	4.5 ± 2.0	(78.6)	4.5 ± 2.1	(73.8)	4.3 ± 2.2	(69.0)	0.0 ± 1.0	−0.2 ± 1.4
(formerly *Lactobacillus gasseri* subgroup)	Synbiotic	5.3 ± 2.0 *	(88.6)	5.9 ± 1.9 **	(93.2) *	6.0 ± 1.9 **	(90.5) *	0.6 ± 1.8	0.6 ± 1.7 *
*Levilactobacillus brevis*	Control	2.1 ± 0.9	(7.1)	2.0 ± 0.6	(2.4)	2.2 ± 1.1	(9.5)	−0.1 ± 1.1	0.1 ± 1.0
(formerly *Lactobacillus brevis*)	Synbiotic	2.2 ± 1.0	(6.8)	2.1 ± 1.0	(2.3)	2.1 ± 0.6	(7.1)	−0.1 ± 1.4	−0.1 ± 1.2
*Lacticaseibacillus*	Control	2.8 ± 1.2	(26.2)	2.6 ± 1.1	(16.7)	2.7 ± 1.2	(21.4)	−0.2 ± 1.4	−0.1 ± 1.2
(formerly *Lactobacillus casei* subgroup)	Synbiotic	3.1 ± 1.5	(31.8)	6.9 ± 0.4 **	(100.0) **	6.9 ± 1.2 **	(95.2) **	3.9 ± 1.5 **	3.8 ± 1.8 **
*Limosilactobacillus fermentum*	Control	3.5 ± 1.7	(26.2)	3.6 ± 1.8	(31.0)	3.4 ± 1.7	(23.8)	0.1 ± 1.2	−0.1 ± 1.4
(formerly *Lactobacillus fermentum*)	Synbiotic	4.1 ± 2.2	(38.6)	4.0 ± 2.2	(34.1)	4.3 ± 2.5	(35.7)	−0.1 ± 1.5	0.2 ± 1.6
*Fructilactobacillus fructivorans*	Control	1.5 ± 0.0	(0.0)	1.5 ± 0.2	(2.4)	1.5 ± 0.0	(0.0)	0.0 ± 0.2	0.0 ± 0.0
(formerly *Lactobacillus fructivorans*)	Synbiotic	1.5 ± 0.3	(2.3)	1.5 ± 0.0	(0.0)	1.5 ± 0.3	(2.4)	0.0 ± 0.3	0.0 ± 0.4
*Lactiplantibacillus*	Control	2.7 ± 1.5	(45.2)	2.7 ± 1.4	(50.0)	3.3 ± 1.6	(69.0)	0.0 ± 1.7	0.6 ± 1.9
(formerly *Lactobacillus plantarum* subgroup)	Synbiotic	3.0 ± 1.6	(54.5)	3.6 ± 1.5 **	(75.0) *	3.3 ± 1.5	(66.7)	0.6 ± 2.2	0.2 ± 2.1
*Limosilactobacillus* (except *L. fermentum*)	Control	3.7 ± 1.8	(50.0)	3.8 ± 1.8	(52.4)	4.1 ± 1.9	(61.9)	0.1 ± 1.1	0.5 ± 1.6
(formerly *Lactobacillus reuteri* subgroup)	Synbiotic	4.5 ± 2.2	(63.6)	5.2 ± 2.0 **	(79.5) **	5.6 ± 1.7 **	(90.5) **	0.7 ± 1.6 *	1.1 ± 1.5
*Ligilactobacillus* and *Liquorilactobacillus*	Control	4.1 ± 2.4	(66.7)	4.0 ± 2.5	(59.5)	4.0 ± 2.5	(57.1)	−0.2 ± 1.3	−0.1 ± 1.6
(formerly *Lactobacillus ruminis* subgroup)	Synbiotic	4.6 ± 2.9	(63.6)	4.6 ± 2.8	(68.2)	4.7 ± 2.8	(69.0)	0.1 ± 1.3	0.1 ± 1.8
*Latilactobacillus*	Control	2.5 ± 1.5	(26.2)	2.4 ± 1.6	(19.0)	2.4 ± 1.7	(19.0)	−0.1 ± 2.4	−0.1 ± 2.1
(formerly *Lactobacillus sakei* subgroup)	Synbiotic	2.8 ± 2.2	(25.0)	2.9 ± 1.8	(34.1)	2.2 ± 1.5	(14.3)	0.1 ± 2.7	−0.6 ± 2.8
*Enterobacteriaceae*	Control	6.6 ± 1.7	(90.5)	6.2 ± 2.0	(81.0)	6.5 ± 1.8	(88.1)	−0.4 ± 2.0	−0.1 ± 1.6
	Synbiotic	6.2 ± 1.9	(84.1)	6.2 ± 1.6	(88.6)	6.5 ± 1.6	(90.5)	0.0 ± 2.0	0.3 ± 1.8
*Enterococcus*	Control	4.8 ± 2.0	(76.2)	4.6 ± 2.1	(69.0)	4.6 ± 1.8	(73.8)	−0.2 ± 2.1	−0.2 ± 2.4
	Synbiotic	4.3 ± 2.3	(59.1)	4.3 ± 2.0	(59.1)	4.5 ± 2.3	(64.3)	−0.1 ± 2.2	0.2 ± 2.7
*Streptococcus*	Control	7.0 ± 2.8	(78.6)	6.5 ± 3.0	(71.4)	6.5 ± 3.0	(71.4)	−0.4 ± 3.5	−0.5 ± 3.5
	Synbiotic	8.2 ± 1.7 *	(95.5)	7.3 ± 2.7	(81.8)	7.6 ± 2.7	(83.3)	−0.9 ± 3.1	−0.7 ± 3.1
*Staphylococcus*	Control	3.9 ± 1.3	(71.4)	3.9 ± 1.5	(66.7)	4.3 ± 1.2	(81.0)	0.1 ± 1.6	0.4 ± 1.5
	Synbiotic	4.2 ± 1.4	(77.3)	4.1 ± 1.4	(72.7)	4.4 ± 1.3	(81.0)	−0.1 ± 1.3	0.2 ± 1.5
*Pseudomonas*	Control	2.4 ± 1.0	(11.9)	2.3 ± 0.9	(11.9)	2.4 ± 0.9	(14.3)	0.0 ± 1.2	0.0 ± 1.2
	Synbiotic	2.3 ± 0.9	(9.1)	2.3 ± 0.9	(9.1)	2.3 ± 1.0	(9.5)	0.0 ± 1.1	0.1 ± 1.2

Data are mean ± SD of bacterial counts (detection ratio %). * *p* < 0.05, ** *p* < 0.01 vs. Control. Each change is expressed as the value measured at 12 and 24 weeks minus the baseline value.

**Table 4:** Relative abundances of phylum levels and their changes as determined by 16S rRNA sequencing.

Phylum		Relative Abundance (%)			Changes (%)	
		0 Weeks	12 Weeks	24 Weeks	12 Weeks	24 Weeks
Actinobacteriota	Control	5.0 ± 10	6.0 ± 14.0	4.7 ± 8.4	1.1 ± 5.6	−0.2 ± 4.2
	Synbiotic	8.2 ± 9.9	20.7 ± 15.4 **	18.7 ± 11.3 **	12.4 ± 13.9 **	10.5 ± 11.1 **
Bacteroidota	Control	47.3 ± 17.5	46.6 ± 19.0	49.0 ± 16.8	−0.7 ± 12.2	1.7 ± 13.6
	Synbiotic	42.6 ± 15.2	36.2 ± 13.6 **	36.2 ± 12.6 **	−6.4 ± 16.1	−6.3 ± 14.5 *
Desulfobacterota	Control	0.15 ± 0.13	0.17 ± 0.28	0.15 ± 0.18	0.02 ± 0.25	0.00 ± 0.16
	Synbiotic	0.20 ± 0.25	0.15 ± 0.17	0.14 ± 0.14	−0.06 ± 0.17	−0.06 ± 0.15
Firmicutes	Control	43.9 ± 16.3	43.5 ± 17.1	40.7 ± 16.2	−0.4 ± 11.5	−3.2 ± 13.3
	Synbiotic	45.6 ± 14.2	41.3 ± 12.8	42.5 ± 12.4	−4.3 ± 12.8	−3.2 ± 14.4
Fusobacteriota	Control	0.96 ± 2.77	0.84 ± 2.63	1.49 ± 3.29	−0.13 ± 1.44	0.53 ± 2.31
	Synbiotic	0.68 ± 1.90	0.07 ± 0.19	0.24 ± 1.07 *	−0.62 ± 1.81	−0.46 ± 1.29 *
Proteobacteria	Control	2.6 ± 3.2	2.7 ± 3.7	3.4 ± 4.3	0.1 ± 2.2	0.8 ± 2.7
	Synbiotic	2.4 ± 3.9	1.5 ± 1.8	2.1 ± 2.7	−0.9 ± 2.9	−0.4 ± 2.6 *
Verrucomicrobiota	Control	0.15 ± 0.44	0.15 ± 0.42	0.44 ± 1.52	0.00 ± 0.59	0.29 ± 1.46
	Synbiotic	0.22 ± 0.68	0.07 ± 0.19	0.11 ± 0.38	−0.16 ± 0.64	−0.12 ± 0.79

Data are mean ± SD of relative abundance (%). * *p* < 0.05, ** *p* < 0.01 vs. Control. Each change is expressed as the value measured at 12 and 24 weeks minus the baseline value.

**Table 5:** Serial changes in microbial diversity in the control and synbiotic groups.

		Measured Values			Changes	
		0 Weeks	12 Weeks	24 Weeks	12 Weeks	24 Weeks
Phylogenic diversity	Control	25.4 ± 6.6	25.7 ± 7.5	24.8 ± 6.1	0.4 ± 3.6	−0.6 ± 5.0
	Synbiotic	26.6 ± 6.3	25.4 ± 6.2	26.2 ± 6.0	−1.3 ± 3.1 *	−0.4 ± 3.1
Observed OTU	Control	210.1 ± 70.4	209.8 ± 74.6	203.4 ± 65.9	−0.3 ± 39.8	−6.8 ± 52.1
	Synbiotic	223.4 ± 65.6	206.5 ± 63.8	217.9 ± 63.4	−16.9 ± 36.9 *	−4.9 ± 43.0
Shannon index	Control	5.9 ± 0.78	5.9 ± 0.88	5.8 ± 0.74	0.0 ± 0.5	−0.0 ± 0.7
	Synbiotic	6.1 ± 0.67	5.8 ± 0.75	6.0 ± 0.65	−0.3 ± 0.5 *	−0.1 ± 0.5

Data are mean ± SD. * *p* < 0.05 vs. Control. Each change is expressed as the value measured at 12 and 24 weeks minus the baseline value.

**Table 6:** Relative abundances at the family level and their changes as determined by 16S rRNA sequencing.

Phylum	Family		Relative Abundance (%)			Changes (%)	
			0 Weeks	12 Weeks	24 Weeks	12 Weeks	24 Weeks
Actinobacteriota	Bifidobacteriaceae	Control	3.9 ± 10.1	4.8 ± 13.7	3.5 ± 8.2	0.9 ± 5.2	−0.4 ± 4.1
		Synbiotic	6.9 ± 9.4	18.9 ± 14.4 **	17.0 ± 10.9 **	12.0 ± 13.3 **	10.1 ± 10.7 **
	Coriobacteriaceae	Control	0.9 ± 0.8	1.1 ± 1.0	1.1 ± 1.0	0.1 ± 1.0	0.1 ± 0.9
		Synbiotic	1.1 ± 1.0	1.6 ± 2.3	1.5 ± 1.0 *	0.4 ± 1.9	0.4 ± 1.1
	Eggerthellaceae	Control	0.10 ± 0.11	0.14 ± 0.21	0.12 ± 0.15	0.04 ± 0.18	0.02 ± 0.13
		Synbiotic	0.11 ± 0.09	0.13 ± 0.11	0.14 ± 0.20	0.02 ± 0.12	0.03 ± 0.22
Bacteroidota	Bacteroidaceae	Control	23.0 ± 17.5	22.4 ± 17.3	24.4 ± 18.4	−0.6 ± 8.1	1.4 ± 11.3
		Synbiotic	25.6 ± 16.0	20.3 ± 13.2	21.4 ± 14.4	−5.3 ± 12.7 *	−4.7 ± 10.8 *
	Barnesiellaceae	Control	0.18 ± 0.32	0.28 ± 0.69	0.23 ± 0.32	0.10 ± 0.65	0.04 ± 0.31
		Synbiotic	0.44 ± 0.55 **	0.35 ± 0.48	0.35 ± 0.40	−0.09 ± 0.44	−0.07 ± 0.42
	Marinifilaceae	Control	0.22 ± 0.21	0.22 ± 0.24	0.29 ± 0.60	0.00 ± 0.24	0.08 ± 0.59
		Synbiotic	0.33 ± 0.40	0.19 ± 0.19	0.24 ± 0.24	−0.14 ± 0.33 *	−0.08 ± 0.28
	Muribaculaceae	Control	0.20 ± 0.41	0.30 ± 0.60	0.18 ± 0.41	0.10 ± 0.48	−0.02 ± 0.35
		Synbiotic	0.22 ± 0.48	0.20 ± 0.40	0.23 ± 0.53	−0.02 ± 0.28	0.01 ± 0.34
	Prevotellaceae	Control	19.4 ± 24.1	20.0 ± 25.7	19.7 ± 24.0	0.6 ± 12.2	0.3 ± 11.8
		Synbiotic	10.8 ± 17.3	11.5 ± 17.3	9.9 ± 15.4 *	0.7 ± 12.4	−0.2 ± 11.6
	Rikenellaceae	Control	1.0 ± 1.4	1.0 ± 1.6	0.8 ± 0.8	−0.1 ± 1.3	−0.3 ± 1.3
		Synbiotic	1.6 ± 2.6	1.2 ± 1.5	1.3 ± 1.6	−0.4 ± 1.6	−0.4 ± 1.6
	Tannerellaceae	Control	3.3 ± 3.0	2.4 ± 2.5	3.5 ± 2.7	−0.8 ± 2.2	0.2 ± 2.8
		Synbiotic	3.6 ± 3.1	2.5 ± 2.6	2.7 ± 2.4	−1.1 ± 1.6	−0.9 ± 2.7
Desulfobacterota	Desulfovibrionaceae	Control	0.15 ± 0.13	0.17 ± 0.28	0.15 ± 0.18	0.02 ± 0.25	0.00 ± 0.16
		Synbiotic	0.20 ± 0.25	0.15 ± 0.17	0.14 ± 0.14	−0.06 ± 0.17	−0.06 ± 0.15
Firmicutes	Acidaminococcaceae	Control	0.61 ± 0.68	0.65 ± 0.89	0.64 ± 0.97	0.03 ± 0.62	0.03 ± 0.75
		Synbiotic	0.99 ± 0.91 *	1.25 ± 1.92	0.96 ± 0.97	0.26 ± 1.63	−0.02 ± 0.73
	Anaerovoracaceae	Control	0.16 ± 0.21	0.23 ± 0.47	0.16 ± 0.30	0.08 ± 0.42	0.00 ± 0.27
		Synbiotic	0.23 ± 0.38	0.17 ± 0.20	0.19 ± 0.22	−0.07 ± 0.31	−0.04 ± 0.22
	Bacillaceae	Control	0.07 ± 0.32	0.14 ± 0.36	0.13 ± 0.35	0.07 ± 0.49	0.06 ± 0.25
		Synbiotic	0.11 ± 0.19	0.09 ± 0.24	0.08 ± 0.24	−0.01 ± 0.24	−0.03 ± 0.3
	Butyricicoccaceae	Control	0.19 ± 0.21	0.18 ± 0.15	0.19 ± 0.21	0.00 ± 0.20	0.00 ± 0.26
		Synbiotic	0.18 ± 0.20	0.15 ± 0.14	0.19 ± 0.15	−0.03 ± 0.17	0.01 ± 0.22
	Christensenellaceae	Control	0.25 ± 0.72	0.46 ± 1.13	0.30 ± 0.73	0.22 ± 0.95	0.06 ± 0.67
		Synbiotic	0.29 ± 0.56	0.19 ± 0.28	0.29 ± 0.54	−0.09 ± 0.42	0.01 ± 0.40
	Clostridiaceae	Control	0.23 ± 0.67	0.13 ± 0.22	0.21 ± 0.80	−0.09 ± 0.68	−0.02 ± 0.9
		Synbiotic	0.13 ± 0.25	0.02 ± 0.04 **	0.14 ± 0.39	−0.12 ± 0.23	0.00 ± 0.36
	Erysipelatoclostridiaceae	Control	1.6 ± 2.4	1.5 ± 2.0	1.3 ± 1.6	−0.1 ± 1.5	−0.3 ± 1.9
		Synbiotic	1.4 ± 2.4	1.2 ± 2.2	1.2 ± 2.2	−0.1 ± 1.6	−0.1 ± 2.0
	Erysipelotrichaceae	Control	2.0 ± 2.8	2.0 ± 2.6	2.2 ± 3.0	0.1 ± 2.7	0.2 ± 2.4
		Synbiotic	1.7 ± 2.7	1.4 ± 2.1	1.4 ± 1.8	−0.3 ± 1.5	−0.4 ± 1.8
	Lachnospiraceae	Control	22.4 ± 11.7	22.1 ± 12.1	20.6 ± 11.2	−0.3 ± 9.7	−1.8 ± 9.3
		Synbiotic	23.4 ± 11.1	19.5 ± 10.4	19.8 ± 10.6	−3.9 ± 9.9	−4.0 ± 9.2
	Lactobacillaceae	Control	0.2 ± 0.8	0.5 ± 2.1	0.5 ± 1.5	0.3 ± 1.4	0.3 ± 1.4
		Synbiotic	1.5 ± 3.1 *	1.5 ± 3.0	1.3 ± 2.4	0.0 ± 2.7	−0.1 ± 2.5
	Monoglobaceae	Control	0.06 ± 0.07	0.19 ± 0.59	0.10 ± 0.13	0.13 ± 0.59	0.04 ± 0.09
		Synbiotic	0.10 ± 0.12	0.08 ± 0.09	0.08 ± 0.07	−0.02 ± 0.11	−0.02 ± 0.12 *
	Oscillospiraceae	Control	1.4 ± 1.7	1.3 ± 1.6	1.0 ± 1.3	−0.1 ± 1.2	−0.3 ± 1.5
		Synbiotic	1.3 ± 1.1	1.1 ± 1.1	1.2 ± 1.2	−0.2 ± 0.9	0.0 ± 1.0
	Peptostreptococcaceae	Control	0.40 ± 0.91	0.39 ± 0.99	0.78 ± 2.75	−0.02 ± 1.00	0.37 ± 1.99
		Synbiotic	0.40 ± 0.96	0.28 ± 0.66	0.26 ± 0.50	−0.12 ± 0.76	−0.14 ± 0.81
	Ruminococcaceae	Control	5.6 ± 4.3	5.6 ± 4.9	4.8 ± 3.7	0.0 ± 3.4	−0.8 ± 3.6
		Synbiotic	6.9 ± 5.3	6.6 ± 5.0	6.9 ± 4.8 *	−0.3 ± 3.6	−0.1 ± 4.7
	Selenomonadaceae	Control	3.0 ± 4.9	2.6 ± 4.4	2.3 ± 4.0	−0.5 ± 3.7	−0.7 ± 2.7
		Synbiotic	1.5 ± 3.3	2.1 ± 4.4	1.6 ± 4.1	0.6 ± 3.3	0.1 ± 3.1
	Streptococcaceae	Control	2.4 ± 6.1	2.2 ± 6.2	2.5 ± 6.8	−0.1 ± 2.0	0.2 ± 3.7
		Synbiotic	2.4 ± 3.6	2.5 ± 5.1	3.4 ± 6.9	0.0 ± 3.9	0.9 ± 5.4
	Veillonellaceae	Control	1.9 ± 1.9	1.9 ± 2.3	1.9 ± 2.3	0.1 ± 2.1	0.0 ± 1.6
		Synbiotic	1.6 ± 1.8	2.2 ± 2.2	2.5 ± 2.6	0.5 ± 1.9	1.0 ± 2.1 *
Fusobacteriota	Fusobacteriaceae	Control	0.96 ± 2.77	0.84 ± 2.63	1.49 ± 3.29	−0.13 ± 1.44	0.53 ± 2.31
		Synbiotic	0.68 ± 1.90	0.07 ± 0.19	0.24 ± 1.07 *	−0.62 ± 1.81	−0.46 ± 1.29 *
Proteobacteria	Enterobacteriaceae	Control	1.5 ± 2.7	1.5 ± 2.9	1.8 ± 3.4	0.0 ± 2.0	0.4 ± 2.3
		Synbiotic	1.4 ± 4.0	0.8 ± 1.7	1.3 ± 2.8	−0.7 ± 2.9	−0.1 ± 3.1
	Succinivibrionaceae	Control	0.33 ± 1.18	0.48 ± 1.76	0.61 ± 2.41	0.15 ± 0.76	0.28 ± 1.52
		Synbiotic	0.15 ± 0.73	0.03 ± 0.07	0.03 ± 0.09	−0.12 ± 0.73	−0.12 ± 0.74
	Sutterellaceae	Control	0.67 ± 0.54	0.67 ± 0.48	0.67 ± 0.53	0.00 ± 0.46	0.00 ± 0.56
		Synbiotic	0.75 ± 0.53	0.60 ± 0.52	0.54 ± 0.44	−0.16 ± 0.51	−0.22 ± 0.57
Verrucomicrobiota	Akkermansiaceae	Control	0.15 ± 0.44	0.15 ± 0.41	0.44 ± 1.51	0.00 ± 0.58	0.28 ± 1.45
		Synbiotic	0.22 ± 0.68	0.06 ± 0.19	0.11 ± 0.38	−0.15 ± 0.64	−0.12 ± 0.79

Data are mean ± SD of relative abundance (%). * *p* < 0.05, ** *p* < 0.01 vs. Control. Each change is expressed as the value measured at 12 and 24 weeks minus the baseline value.

**Table 7:** Serial changes in fecal organic acids and pH.

		Fecal Organic Acids (µmol/g Feces)						Changes	
		0 Weeks		12 Weeks		24 Weeks		12 Weeks	24 Weeks
Total organic acids	Control	96.5 ± 32.9	(100.0)	91.8 ± 27.1	(100.0)	91.7 ± 24.8	(100.0)	−4.8 ± 37.9	−4.9 ± 32.4
	Synbiotic	80.4 ± 41.5	(100.0)	87.1 ± 30.3	(100.0)	96.8 ± 40.1	(100.0)	5.2 ± 43.5	15.3 ± 50.7 *
Acetic acid	Control	55.2 ± 18.6	(100.0)	52.2 ± 17.0	(100.0)	54.5 ± 17.3	(100.0)	−3.0 ± 23.7	−0.7 ± 20.7
	Synbiotic	49.5 ± 27.7	(100.0)	54.1 ± 21.8	(100.0)	61.9 ± 28.3	(100.0)	3.8 ± 27.7	11.9 ± 34.2 *
Propionic acid	Control	21.8 ± 11.6	(100.0)	21.3 ± 10.8	(100.0)	21.3 ± 10.7	(100.0)	−0.5 ± 11.9	−0.5 ± 10.6
	Synbiotic	16.5 ± 8.5 *	(100.0)	17.2 ± 6.4 *	(100.0)	18.6 ± 9.4	(100.0)	0.3 ± 9.7	1.7 ± 10.9
Butyric acid	Control	10.9 ± 8.1	(97.6)	10.4 ± 5.2	(100.0)	8.0 ± 4.5	(100.0)	−0.5 ± 7.7	−2.9 ± 8.5
	Synbiotic	8.2 ± 7.8	(93.0)	8.7 ± 4.5	(97.7)	9.5 ± 7.7	(100.0)	0.5 ± 8.9	1.3 ± 9.8 *
Isovaleric acid	Control	2.3 ± 2.3	(69.0)	2.4 ± 2.2	(76.2)	2.1 ± 1.9	(71.4)	0.1 ± 2.0	−0.2 ± 2.0
	Synbiotic	2.2 ± 1.5	(81.4)	1.8 ± 1.4	(70.5)	2.2 ± 1.6	(79.1)	−0.4 ± 2.0	0.0 ± 1.9
Valeric acid	Control	2.3 ± 2.0	(71.4)	2.6 ± 2.0	(81.0)	2.4 ± 1.8	(83.3)	0.3 ± 1.7	0.1 ± 2.1
	Synbiotic	1.9 ± 1.4	(72.1)	2.2 ± 1.4	(84.1)	2.2 ± 1.7	(86.0)	0.3 ± 1.6	0.4 ± 2.1
Succinic acid	Control	2.5 ± 6.7	(78.6)	1.8 ± 4.0	(83.3)	2.3 ± 5.3	(81.0)	−0.7 ± 5.3	−0.3 ± 3.2
	Synbiotic	1.4 ± 3.3	(79.1)	1.5 ± 4.7	(86.4)	1.3 ± 1.8	(93.0)	0.2 ± 6.0	−0.3 ± 2.8
Formic acid	Control	0.9 ± 1.6	(66.7)	1.0 ± 1.6	(78.6)	0.7 ± 1.0	(76.2)	0.1 ± 1.0	−0.2 ± 1.0
	Synbiotic	0.8 ± 1.8	(79.1)	0.7 ± 1.1	(75.0)	0.7 ± 0.7	(72.1)	−0.1 ± 1.8	−0.1 ± 1.9
Lactic acid	Control	1.0 ± 2.8	(31.0)	0.3 ± 0.5	(21.4)	0.7 ± 2.5	(26.2)	−0.7 ± 2.8	−0.3 ± 3.8
	Synbiotic	0.3 ± 0.4	(30.2)	1.0 ± 2.9	(36.4)	0.6 ± 1.0	(30.2)	0.7 ± 3.0 *	0.3 ± 1.0
pH	Control	6.6 ± 0.6	(100.0)	6.4 ± 0.5	(100.0)	6.5 ± 0.6	(100.0)	−0.2 ± 0.8	−0.1 ± 0.8
	Synbiotic	6.8 ± 0.5	(100.0)	6.6 ± 0.6	(100.0)	6.5 ± 0.6	(100.0)	−0.2 ± 0.7	−0.3 ± 0.6

Data are mean ± SD of fecal organic acids (detection ratio %). * *p* < 0.05 vs. Control. Each change is expressed as the value measured at 12 and 24 weeks minus baseline value.

## Supplementary Materials

The following are available online at https://www.mdpi.com/2072-6643/13/2/558/s1, Table S1: Primers used in this study, Table S2: Bacterial counts and detection rates of *Lacticaseibacillus paracasei* strain Shirota and *B. breve* strain Yakult in feces determined by qPCR, Table S3: Relative abundance at the species level and their changes as determined by 16S rRNA sequencing, Table S4: Adverse events and changes in diabetes treatment.

## References

[1] Cani P.D., Bibiloni R., Knauf C., Waget A., Neyrinck A.M., Delzenne N.M., Burcelin R. (2008). Changes in gut microbiota control metabolic endotoxemia-induced inflammation in high-fat diet-induced obesity and diabetes in mice. Diabetes.

[2] Pedersen H.K., Gudmundsdottir V., Nielsen H.B., Hyotylainen T., Nielsen T., Jensen B.A.H., Forslund K., Hildebrand F., Prifti E., Falony G. (2016). Human gut microbes impact host serum metabolome and insulin sensitivity. Nature.

[3] De Vadder F., Kovatcheva-Datchary P., Goncalves D., Vinera J., Zitoun C., Duchampt A., Bäckhed F., Mithieux G. (2014). Microbiota-generated metabolites promote metabolic benefits via gut-brain neural circuits. Cell.

[4] Tolhurst G., Heffron H., Lam Y.S., Parker H.E., Habib A.M., Diakogiannaki E., Cameron J., Grosse J., Reimann F., Gribble F.M. (2012). Short-chain fatty acids stimulate glucagon-like peptide-1 secretion via the G-protein-coupled receptor FFAR2. Diabetes.

[5] Miyamoto J., Igarashi M., Watanabe K., Karaki S.-I., Mukouyama H., Kishino S., Li X., Ichimura A., Irie J., Sugimoto Y. (2019). Gut microbiota confers host resistance to obesity by metabolizing dietary polyunsaturated fatty acids. Nat. Commun..

[6] Kimura I., Inoue D., Maeda T., Hara T., Ichimura A., Miyauchi S., Kobayashi M., Hirasawa A., Tsujimoto G. (2011). Short-chain fatty acids and ketones directly regulate sympathetic nervous system via G protein-coupled receptor 41 (GPR41). Proc. Natl. Acad. Sci. USA.

[7] Sato J., Kanazawa A., Ikeda F., Yoshihara T., Goto H., Abe H., Komiya K., Kawahuchi M., Shimizu T., Ogihara T. (2014). Gut dysbiosis and detection of “live gut bacteria” in blood of Japanese patients with type 2 diabetes. Diabetes Care.

[8] Sato J., Kanazawa A., Azuma K., Ikeda F., Goto H., Komiya K., Kanno R., Tamura Y., Asahara T., Takahashi T. (2017). Probiotic reduces bacterial translocation in type 2 diabetes mellitus: A randomised controlled study. Sci. Rep..

[9] Cani P.D., Amar J., Iglesias M.A., Poggi M., Knauf C., Bastelica D., Neyrinck A.M., Fava F., Tuohy K.M., Chabo C. (2007). Metabolic endotoxemia initiates obesity and insulin resistance. Diabetes.

[10] Mehta N.N., McGillicuddy F.C., Anderson P.D., Hinkle C.C., Shah R., Pruscino L., Tabita-Martinez J., Sellers K.F., Rickels M.R., Reilly M.P. (2010). Experimental endotoxemia induces adipose inflammation and insulin resistance in humans. Diabetes.

[11] Liu X., Lu L., Yao P., Ma Y., Wang F., Jin Q., Ye X., Li H., Hu F.B., Sun L. (2014). Lipopolysaccharide binding protein, obesity status and incidence of metabolic syndrome: A prospective study among middle-aged and older Chinese. Diabetologia.

[12] Moreno-Navarrete J.M., Ortega F., Serino M., Luche E., Pardo G., Salvador J., Ricart W., Frühbeck G., Burcelin R., Fernández-Real J.M. (2012). Circulating lipopolysaccharide-binding protein (LBP) as a marker of obesity-related insulin resistance. Int. J. Obes..

[13] Tamaki S., Kanazawa A., Sato J., Tamura Y., Asahara T., Takahashi T., Matsumoto S., Yamashiro Y., Watada H. (2019). Clinical factors associated with bacterial translocation in Japanese patients with type 2 diabetes: A retrospective study. PLoS ONE.

[14] Tenorio-Jimenez C., Martinez-Ramirez M.J., Gil A., Gomez-Llorente C. (2020). Effects of Probiotics on Metabolic Syndrome: A Systematic Review of Randomized Clinical Trials. Nutrients.

[15] Sugawara G., Nagino M., Nishio H., Ebata T., Takagi K., Asahara T., Nomoto K., Nimura Y. (2006). Perioperative synbiotic treatment to prevent postoperative infectious complications in biliary cancer surgery: A randomized controlled trial. Ann. Surg..

[16] Matsuda K., Tsuji H., Asahara T., Kado Y., Nomoto K. (2007). Sensitive quantitative detection of commensal bacteria by rRNA-targeted reverse transcription-PCR. Appl. Environ. Microbiol..

[17] Matsuda K., Tsuji H., Asahara T., Matsumoto K., Takada T., Nomoto K. (2009). Establishment of an analytical system for the human fecal microbiota, based on reverse transcription-quantitative PCR targeting of multicopy rRNA molecules. Appl. Environ. Microbiol..

[18] Sakaguchi S., Saito M., Tsuji H., Asahara T., Takata O., Fujimura J., Nagata S., Nomoto K., Shimizu T. (2010). Bacterial rRNA-targeted reverse transcription-PCR used to identify pathogens responsible for fever with neutropenia. J. Clin. Microbiol..

[19] Ohigashi S., Sudo K., Kobayashi D., Takahashi T., Asahara T., Nomoto K., Onodera H. (2013). Changes of the intestinal microbiota, short chain fatty acids, and fecal pH in patients with colorectal cancer. Dig. Dis. Sci..

[20] Fujimoto J., Tanigawa K., Kudo Y., Makino H., Watanabe K. (2011). Identification and quantification of viable Bifidobacterium breve strain Yakult in human faeces by using strain-specific primers and propidium monoazide. J. Appl. Microbiol..

[21] Fujimoto J., Matsuki T., Sasamoto M., Tomii Y., Watanabe K. (2008). Identification and quantification of *Lactobacillus casei* strain Shirota in human feces with strain-specific primers derived from randomly amplified polymorphic DNA. Int. J. Food Microbiol..

[22] Matsuki T., Watanabe K., Fujimoto J., Takada T., Tanaka R. (2004). Use of 16S rRNA gene-targeted group-specific primers for real-time PCR analysis of predominant bacteria in human feces. Appl. Environ. Microbiol..

[23] Gonai M., Shigehisa A., Kigawa I., Kurasaki A., Chonan O., Matsuki T., Yoshida Y., Aida M., Hamano K., Terauchi Y. (2017). Galacto-oligosaccharides ameliorate dysbiotic Bifidobacteriaceae decline in Japanese patients with type 2 diabetes. Benef. Microbes.

[24] Ikeda T., Aida M., Yoshida Y., Matsumoto S., Tanaka M., Nakayama J., Nagao Y., Nakata R., Oki E., Akahoshi T. (2020). Alteration in faecal bile acids, gut microbial composition and diversity after laparoscopic sleeve gastrectomy. Br. J. Surg..

[25] Bolyen E., Rideout J.R., Dillon M.R., Bokulich N.A., Abnet C.C., Al-Ghalith G.A., Alexander H., Alm E.J., Arumugam M., Asnicar F. (2019). Reproducible, interactive, scalable and extensible microbiome data science using QIIME 2. Nat. Biotechnol..

[26] Segata N., Izard J., Waldron L., Gevers D., Miropolsky L., Garrett W.S., Huttenhower C. (2011). Metagenomic biomarker discovery and explanation. Genome Biol..

[27] Kobyliak N., Falalyeyeva T., Mykhalchyshyn G., Kyriienko D., Komissarenko I. (2018). Effect of alive probiotic on insulin resistance in type 2 diabetes patients: Randomized clinical trial. Diabetes Metab. Syndr..

[28] Kobyliak N., Abenavoli L., Mykhalchyshyn G., Kononenko L., Boccuto L., Kyriienko D., Dynnyk O. (2018). A Multi-strain Probiotic Reduces the Fatty Liver Index, Cytokines and Aminotransferase levels in NAFLD Patients: Evidence from a Randomized Clinical Trial. J. Gastrointest. Liver Dis..

[29] Tonucci L.B., Olbrich Dos Santos K.M., Licursi de Oliveira L., Rocha Ribeiro S.M., Duarte Martino H.S. (2017). Clinical application of probiotics in type 2 diabetes mellitus: A randomized, double-blind, placebo-controlled study. Clin. Nutr..

[30] Mohamadshahi M., Veissi M., Haidari F., Shahbazian H., Kaydani G.A., Mohammadi F. (2014). Effects of probiotic yogurt consumption on inflammatory biomarkers in patients with type 2 diabetes. Bioimpacts.

[31] Wu H., Esteve E., Tremaroli V., Khan M.T., Caesar R., Mannerås-Holm L., Ståhlman M., Olsson L.M., Planas-Fèlix M., Xifra G. (2017). Metformin alters the gut microbiome of individuals with treatment-naive type 2 diabetes, contributing to the therapeutic effects of the drug. Nat. Med..

[32] Cornejo-Pareja I., Martín-Núñez G.M., Roca-Rodríguez M.M., Cardona F., Coin-Aragüez L., Sánchez-Alcoholado L., Gutiérrez-Repiso C., Muñoz-Garach A., Fernández-García J.C., Moreno-Indias I. (2019). H. pylori Eradication Treatment Alters Gut Microbiota and GLP-1 Secretion in Humans. J. Clin. Med..

[33] Zeng Z., Luo J.Y., Zuo F.L., Yu R., Zhang Y., Ma H.Q., Chen S.W. (2016). Bifidobacteria possess inhibitory activity against dipeptidyl peptidase-IV. Lett. Appl. Microbiol..

[34] Sanchis-Chordà J., Del Pulgar E.M.G., Carrasco-Luna J., Benítez-Páez A., Sanz Y., Codoñer-Franch P. (2019). Bifidobacterium pseudocatenulatum CECT 7765 supplementation improves inflammatory status in insulin-resistant obese children. Eur. J. Nutr..

[35] Cano P.G., Santacruz A., Trejo F.M., Sanz Y. (2013). Bifidobacterium CECT 7765 improves metabolic and immunological alterations associated with obesity in high-fat diet-fed mice. Obesity (Silver Spring).

[36] Ruiz-Aceituno L., Esteban-Torres M., James K., Moreno F.J., van Sinderen D. (2020). Metabolism of biosynthetic oligosaccharides by human-derived *Bifidobacterium breve* UCC2003 and *Bifidobacterium longum* NCIMB 8809. Int. J. Food Microbiol..

[37] Matsuki T., Pédron T., Regnault B., Mulet C., Hara T., Sansonetti P.J. (2013). Epithelial cell proliferation arrest induced by lactate and acetate from *Lactobacillus casei* and *Bifidobacterium breve*. PLoS ONE.

[38] Wan Saudi W.S., Sjöblom M. (2017). Short-chain fatty acids augment rat duodenal mucosal barrier function. Exp. Physiol..

[39] Kimura I., Ozawa K., Inoue D., Imamura T., Kimura K., Maeda T., Terasawa K., Kashihara D., Hirano K., Tani T. (2013). The gut microbiota suppresses insulin-mediated fat accumulation via the short-chain fatty acid receptor GPR43. Nat. Commun..

[40] Nagpal R., Tsuji H., Takahashi T., Kawashima K., Nagata S., Nomoto K., Yamashiro Y. (2016). Sensitive Quantitative Analysis of the Meconium Bacterial Microbiota in Healthy Term Infants Born Vaginally or by Cesarean Section. Front. Microbiol..

[41] Maathuis A.J., van den Heuvel E.G., Schoterman M.H., Venema K. (2012). Galacto-oligosaccharides have prebiotic activity in a dynamic in vitro colon model using a (13)C-labeling technique. J. Nutr..

[42] Selle K., Klaenhammer T.R. (2013). Genomic and phenotypic evidence for probiotic influences of *Lactobacillus gasseri* on human health. FEMS Microbiol. Rev..

[43] Karlsson F.H., Tremaroli V., Nookaew I., Bergström G., Behre C.J., Fagerberg B., Nielsen J., Bäckhed F. (2013). Gut metagenome in European women with normal, impaired and diabetic glucose control. Nature.

[44] Sicard J.F., Le Bihan G., Vogeleer P., Jacques M., Harel J. (2017). Interactions of Intestinal Bacteria with Components of the Intestinal Mucus. Front. Cell Infect. Microbiol..

[45] Corridoni D., Pastorelli L., Mattioli B., Locovei S., Ishikawa D., Arseneau K.O., Chieppa M., Cominelli F., Pizarro T.T. (2012). Probiotic bacteria regulate intestinal epithelial permeability in experimental ileitis by a TNF-dependent mechanism. PLoS ONE.

