# Supplementary material for: Effects of Synbiotic Supplementation on Chronic Inflammation and the Gut Microbiota in Obese Patients with Type 2 Diabetes Mellitus: A Randomized Controlled Study
Source: Nutrients. 2021 Feb 8;13(2):558. doi: 10.3390/nu13020558 (PMC7914668; doi:10.3390/nu13020558)
Supplement: Supplementary file 1 [file nutrients-13-00558-s001.pdf]

nutrients

MDPI

Supplementary table

Effects of Synbiotic Supplementation on Chronic Inflammation and the Gut Microbiota in Obese Patients with Type 2 Diabetes Mellitus: a Randomized Controlled Study

Akio Kanazawa[1*], Masanori Aida[6], Yasuto Yoshida[6], Hideyoshi Kaga[1], Takehiro Katahira[1], Luka Suzuki[1], Shoko Tamaki[1], Junko Sato[1], Hiromasa Goto[1], Kosuke Azuma[1], Tomoaki Shimizu[1], Takuya Takahashi[7], Yuichiro Yamashiro[5] and Hirotaka Watada[1,2,3,4]

Table S1. Primers used in this study.

Target bacteria*	Primer	Sequence (5' - 3')	Ref
Clostridium coccoides group	g-Ccoc-F	AAATGACGGTACCTGACTAA	5
	g-Ccoc-R	CTTTGAGTTTCATTCTTGCGAA	
Clostridium leptum subgroup	sg-Clept-F	GCACAAGCAGTGGAGT	6
	sg-Clept-R3	CTTCCTCCGTTTTGTCAA	
Bacteroides fragilis group	g-Bfra-F2	AYAGCCTTTCGAAAGRAAGAT	7
	g-Bfra-R	CCAGTATCAACTGCAATTTTA	5
Bifidobacterium	g-Bifid-F	CTCCTGGAAACGGGTGG	5
	g-Bifid-R	GGTGTTCTTCCCGATATCTACA	
Atopobium cluster	g-Atopo-F	GGGTTGAGAGACCGACC	6
	g-Atopo-R	CGGRGCTTCTTCTGCAGG	
Prevotella	g-Prevo-F	CACRGTAAACGATGGATGCC	5
	g-Prevo-R	GGTCGGGTTGCAGACC	
Akkermansia muciniphila	AM1	CAGCACGTGAAGGTGGGGAC	8
	AM2	CCTTGCGGTTGGCTTCAGAT	
Clostridium perfringens	s-Clper-F	GGGGGTTTCAACACCTCC	2
	ClPER-R	GCAAGGGATGTCAAGTGT	5
Lactobacillus (formerly *Lactobacillus gasseri* subgroup)	sg-Lgas-F	GATGCATAGCCGAGTTGAGAGACTGAT	2
	sg-Lgas-R	TAAAGGCCAGTTACTACCTCTATCC	
Levilactobacillusbrevis (formerly *Lactobacillus brevis*)	s-Lbre-F	ATTTTGTTTGAAAGGTGGCTTCGG	2
	s-Lbre-R	ACCCTTGAACAGTTACTCTCAAAGG	
Lacticaseibacillus (formerly *Lactobacillus casei* subgroup)	sg-Lcas-F	ACCGCATGGTTCTTGGC	2
	sg-Lcas-R	CCGACAACAGTTACTCTGCC	
Limosilactobacillus fermentum (formerly *Lactobacillus fermentum*)	LFer-1	CCTGATTGATTTTGGTCGCCAAC	2
	LFer-2	ACGTATGAACAGTTACTCTCATACGT	
Fructilactobacillus fructiborans (formerly *Lactobacillus fructiborans*)	s-Lfru-F	TGCGCCTAATGATAGTTGA	2
	s-Lfru-R	GATACCGTCGCGACGTGAG	
Lactiplantibacillus (formerly *Lactobacillus plantarum* subgroup)	sg-Lpla-F	CTCTGGTATTGATTGGTGCTTGCAT	2
	sg-Lpla-R	GTTCGCCACTCACTCAAATGTAAA	
Limosilactobacillus (except *L. fermentum*) (formerly *Lactobacillus reuteri* subgroup)	sg-Lreu-F	GAACGCAYTGGCCCAA	2
	sg-Lreu-R	TCCATTGTGGCCGATCAGT	
Ligilactobacillus and *Liquorilactobacillus* (formerly *Lactobacillus ruminis* subgroup)	sg-Lrum-F	CACCGAATGCTTGCAYTCACC	2
	sg-Lrum-R	GCCGCGGGTCCATCCAAAA	
Latilactobacillus (formerly *Lactobacillus sakei* subgroup)	sg-Lsak-F	CATAAAACCTAMCACCGCATGG	2
	sg-Lsak-R	TCAGTTACTATCAGATACRTTCTTCTC	
Enterobacteriaceae	En-lsu-3F	TGCCGTAACTTCGGGAGAAGGCA	1
	En-lsu-3'R	TCAAGGACCAGTGTTCAGTGTC	
Enterococcus	g-Encoc-F	ATCAGAGGGGGATAACACTT	2
	g-Encoc-R	ACTCTCATCCTTGTTCTTCTC	
Streptococcus	g-Str-F	AGCTTAGAAGCAGCTATTCATTC	3
	g-Str-R	GGATACACCTTTCGGTCTCTC	
Staphylococcus	g-Staph-F	TTTGGGCTACACACGTGCTACAATGGACAA	2
	g-Staph-R	AACAACTTTATGGGATTTGCWTGA	

Pseudomonas	PSD7F	CAAAACTACTGAGCTAGAGTACG	1
	PSD7R	TAAGATCTCAAGGATCCCAACGGCT	
Lacticaseibacillus paracasei strain Shirota[$]	pLcS-57F	CTCAAAGCCGTGACGGTC	9
	pLcS-597R	ACGTGGTGCTAATAATCCTAGTG	
Bifidobacterium breve strain Yakult[$]	pBbrY-F	ATGGCAAAACCGGGCTGAA	10
	pBbrY-R	GCGGATGAGAGGTGGG	

* Group-, genus- or species-specific primer sets were developed using 16S rDNA sequences, except for En-lsu-3F/3'R, and g-Str-F/R, which targeted 23S rDNA.

[$] Strain-specific primers sets for *Lacticaseibacillus paracasei* strain Shirota and *Bifidobacterium breve* strain Yakult were used.

.

Table S2. Bacterial counts and detection rates of *Lacticaseibacillus paracasei* strain Shirota and *B. breve* strain Yakult in feces determined by qPCR.

		**Fecal organic acids (µmol/g feces)**						**Changes**	
		**0 weeks**		**12 weeks**		**24 weeks**		**12 weeks**	**24 weeks**
Lacticaseibacillus paracasei strain Shirota	Control	6.5	(2.4)	7.7	(2.4)	<5.9	(0.0)	0.0 ± 0.9	-0.1 ± 0.6
	Synbiotic	8.1 ± 0.9	(4.5)	7.9 ± 0.1**	(97.7)**	8.1 ± 0.3**	(92.9)**	4.6 ± 1.4**	4.6 ± 1.8**
B. breve strain Yakult	Control	<6.1	(0.0)	7.3	(2.4)	<6.1	(0.0)	0.1 ± 0.6	0.0 ± 0.0
	Synbiotic	8.6 ± 0.1	(4.5)	8.1 ± 1.1*	(18.2)*	8.3 ± 0.8**	(19.1)**	0.7 ± 2.1	0.7 ± 2.5

Data are mean ± SD of bacterial counts (detection ratio %). * $p < 0.05$, ** $p < 0.01$ vs. Control. Each change is expressed as the value measured at 12 and 24 weeks minus baseline value.

Table S3. Relative abundance at the species level and their changes as determined by 16S rRNA sequencing.

Phylum	Family	Species		Relative abundance (%)			Changes (%)	
				0 weeks	12 weeks	24 weeks	12 weeks	24 weeks
Actinobacteriota	*Bifidobacteriaceae*	*Bifidobacterium pseudocatenulatum*	Control	1.3 ± 3.4	1.9 ± 5.7	0.9 ± 2.2	0.6 ± 3.0	-0.3 ± 1.8
			Synbiotic	2.1 ± 5.1	6.3 ± 9.4*	5.4 ± 6.7**	4.2 ± 8.8*	3.2 ± 6.8**
		Bifidobacterium kashiwanohense	Control	0.86 ± 5.58	0.88 ± 5.73	0.50 ± 3.25	0.02 ± 0.15	-0.36 ± 2.32
			Synbiotic	0.00 ± 0.02	0.01 ± 0.08	0.01 ± 0.03	0.01 ± 0.06	0.00 ± 0.01
		Bifidobacterium adolescentis	Control	0.8 ± 1.7	1.0 ± 2.9	0.8 ± 1.7	0.2 ± 3.0	-0.1 ± 2.2
			Synbiotic	3.3 ± 5.3**	8.7 ± 10.4**	7.4 ± 8.6**	5.4 ± 9.1**	4.1 ± 7.3**
	Coriobacteriaceae	*Collinsella aerofaciens*	Control	0.49 ± 0.52	0.55 ± 0.81	0.51 ± 0.48	0.06 ± 0.8	0.01 ± 0.57
			Synbiotic	0.59 ± 0.75	0.64 ± 0.73	0.72 ± 0.62	0.05 ± 0.38	0.14 ± 0.57
Bacteroidota	*Bacteroidaceae*	*Bacteroides vulgatus*	Control	0.06 ± 0.33	0.03 ± 0.20	0.09 ± 0.47	-0.02 ± 0.13	0.03 ± 0.15
			Synbiotic	0.39 ± 1.16	0.28 ± 1.12	0.26 ± 0.91	-0.11 ± 0.56	-0.14 ± 0.38**
		Bacteroides uniformis	Control	1.8 ± 2.4	1.6 ± 2.3	1.8 ± 2.3	-0.2 ± 2.6	-0.1 ± 1.6
			Synbiotic	2.7 ± 3.0	1.6 ± 2.0	2.1 ± 2.8	-1.1 ± 2.2	-0.7 ± 2.2
		Bacteroides thetaiotaomicron	Control	0.33 ± 0.47	0.27 ± 0.33	0.30 ± 0.37	-0.06 ± 0.49	-0.03 ± 0.42
			Synbiotic	0.53 ± 0.55	0.42 ± 0.58	0.42 ± 0.65	-0.11 ± 0.48	-0.11 ± 0.56
		Bacteroides stercoris	Control	2.2 ± 3.9	2.4 ± 4.3	2.4 ± 5.7	0.3 ± 2.2	0.2 ± 3.4
			Synbiotic	2.0 ± 3.5	1.4 ± 2.0	1.8 ± 3.5	-0.6 ± 2.3	-0.2 ± 2.0
		Bacteroides plebeius	Control	3.4 ± 9.8	3.2 ± 8.3	4.2 ± 10.4	-0.2 ± 3.4	0.8 ± 4.1
			Synbiotic	2.4 ± 6.1	3.6 ± 7.2	2.3 ± 4.9	1.3 ± 5.0	-0.2 ± 3.7
		Bacteroides massiliensis	Control	1.5 ± 4.6	1.2 ± 2.7	1.0 ± 2.5	-0.3 ± 2.7	-0.5 ± 2.8
			Synbiotic	0.8 ± 2.2	0.5 ± 1.4	0.5 ± 1.5	-0.3 ± 1.9	-0.3 ± 1.2
		Bacteroides eggerthii	Control	0.15 ± 0.58	0.11 ± 0.28	0.04 ± 0.10	-0.04 ± 0.56	-0.11 ± 0.56
			Synbiotic	0.37 ± 1.12	0.31 ± 0.94	0.23 ± 0.77	-0.06 ± 0.54	-0.15 ± 0.49
		Bacteroides dorei	Control	1.0 ± 3.9	1.1 ± 3.9	1.0 ± 3.4	0.1 ± 0.4	0.0 ± 0.7
			Synbiotic	1.3 ± 3.4	1.1 ± 2.9	1.2 ± 3.0	-0.3 ± 1.1	-0.1 ± 1.4
		Bacteroides coprophilus	Control	0.17 ± 0.79	0.13 ± 0.60	0.16 ± 0.73	-0.04 ± 0.26	-0.01 ± 0.08
			Synbiotic	0.52 ± 2.06	0.27 ± 1.00	0.24 ± 1.15	-0.25 ± 1.55	-0.29 ± 1.83
		Bacteroides coprocola	Control	1.9 ± 4.9	1.6 ± 4.5	1.3 ± 3.6	-0.3 ± 1.4	-0.6 ± 1.7
			Synbiotic	0.4 ± 1.6*	0.5 ± 2.3	0.4 ± 2.1	0.1 ± 0.7	0.0 ± 0.6*
		Bacteroides cellulosilyticus	Control	0.12 ± 0.33	0.18 ± 0.44	0.47 ± 1.54	0.06 ± 0.37	0.35 ± 1.49
			Synbiotic	0.11 ± 0.26	0.12 ± 0.40	0.10 ± 0.32	0.01 ± 0.32	-0.01 ± 0.27
		Bacteroides caccae	Control	0.28 ± 0.51	0.38 ± 0.64	0.26 ± 0.44	0.10 ± 0.63	-0.02 ± 0.38
			Synbiotic	0.45 ± 0.66	0.30 ± 0.44	0.51 ± 0.88	-0.15 ± 0.4*	0.05 ± 0.51
	Marinifilaceae	*Odoribacter splanchnicus*	Control	0.15 ± 0.15	0.12 ± 0.11	0.12 ± 0.13	-0.03 ± 0.14	-0.03 ± 0.14
			Synbiotic	0.14 ± 0.15	0.1 ± 0.11	0.12 ± 0.14	-0.04 ± 0.12	-0.02 ± 0.14

	Prevotellaceae	*Prevotella stercorea*	Control	0.58 ± 1.50	0.59 ± 1.34	0.47 ± 1.11	0.01 ± 0.74	-0.10 ± 0.79
			Synbiotic	0.54 ± 1.85	0.69 ± 2.31	0.28 ± 0.80	0.15 ± 0.81	-0.26 ± 1.58
		Prevotella copri	Control	1.3 ± 3.5	2.1 ± 5.5	1.5 ± 4.2	0.9 ± 3.1	0.3 ± 1.9
			Synbiotic	0.9 ± 2.6	0.7 ± 1.8	0.7 ± 1.7	-0.2 ± 2.4	0.0 ± 1.7
	Rikenellaceae	*Alistipes onderdonkii*	Control	0.06 ± 0.17	0.08 ± 0.31	0.05 ± 0.15	0.02 ± 0.33	-0.01 ± 0.09
			Synbiotic	0.17 ± 0.41	0.15 ± 0.45	0.14 ± 0.27	-0.02 ± 0.40	-0.04 ± 0.36
	Tannerellaceae	*Parabacteroides merdae*	Control	0.78 ± 1.13	0.7 ± 0.98	0.95 ± 1.36	-0.09 ± 0.66	0.17 ± 1.22
			Synbiotic	0.96 ± 1.27	0.64 ± 0.6	0.89 ± 1.03	-0.32 ± 1.02	-0.04 ± 1.07
Firmicutes	*Acidaminococcaceae*	*Phascolarctobacterium faecium*	Control	0.14 ± 0.32	0.18 ± 0.44	0.19 ± 0.56	0.03 ± 0.29	0.05 ± 0.38
			Synbiotic	0.30 ± 0.51	0.21 ± 0.40	0.26 ± 0.54	-0.09 ± 0.26*	-0.04 ± 0.40
	Erysipelotrichaceae	*Chlamydia trachomatis*	Control	0.12 ± 0.58	0.11 ± 0.50	0.22 ± 1.01	-0.01 ± 0.27	0.09 ± 0.45
			Synbiotic	0.07 ± 0.50	0.04 ± 0.26	0.11 ± 0.71	-0.04 ± 0.23	0.03 ± 0.21
	Lachnospiraceae	*Eubacterium hallii*	Control	1.2 ± 2.5	1.2 ± 2.6	1.4 ± 4.3	0.0 ± 0.8	0.2 ± 2.1
			Synbiotic	0.7 ± 1.1	0.4 ± 0.6	0.5 ± 0.7	-0.3 ± 0.8	-0.2 ± 0.6
		Eubacterium rectale	Control	1.7 ± 2.6	1.9 ± 3.5	1.7 ± 3.4	0.2 ± 2.8	0.0 ± 2.3
			Synbiotic	2.7 ± 4.1	2.0 ± 2.5	2.3 ± 3.8	-0.8 ± 2.8	-0.5 ± 2.7
		Anaerostipes hadrus	Control	1.2 ± 1.7	1.9 ± 4.3	1.4 ± 2.6	0.8 ± 3.5	0.3 ± 1.6
			Synbiotic	1.6 ± 2.0	1.7 ± 2.4	1.5 ± 2.2	0.1 ± 2.2	-0.2 ± 1.8
		Dorea formicigenerans	Control	0.24 ± 0.23	0.24 ± 0.26	0.20 ± 0.16	0.01 ± 0.25	-0.04 ± 0.26
			Synbiotic	0.16 ± 0.19	0.20 ± 0.30	0.16 ± 0.18	0.04 ± 0.25	0.00 ± 0.18
		Roseburia inulinivorans	Control	0.35 ± 0.58	0.35 ± 0.52	0.34 ± 0.55	0.00 ± 0.35	-0.01 ± 0.58
			Synbiotic	0.34 ± 0.65	0.13 ± 0.32*	0.17 ± 0.32	-0.21 ± 0.38**	-0.17 ± 0.44
	Lactobacillaceae	*Lactobacillus salivarius*	Control	0.04 ± 0.17	0.11 ± 0.49	0.07 ± 0.25	0.07 ± 0.34	0.03 ± 0.22
		(now *Ligilactobacillus salivarius*)	Synbiotic	0.18 ± 0.63	0.17 ± 0.77	0.14 ± 0.59	0.00 ± 0.35	-0.04 ± 0.33
		Lactobacillus mucosae	Control	0.03 ± 0.15	0.10 ± 0.44	0.11 ± 0.48	0.07 ± 0.33	0.08 ± 0.46
		(now *Limosilactobacillus mucosae*)	Synbiotic	0.20 ± 0.58	0.18 ± 0.48	0.29 ± 0.88	-0.02 ± 0.58	0.08 ± 0.73
	Ruminococcaceae	*Faecalibacterium prausnitzii*	Control	0.39 ± 0.59	0.38 ± 0.60	0.25 ± 0.41	-0.01 ± 0.38	-0.15 ± 0.30
			Synbiotic	0.35 ± 0.56	0.24 ± 0.38	0.28 ± 0.43	-0.11 ± 0.43	-0.08 ± 0.52
		Ruminococcus bicirculans	Control	0.30 ± 0.79	0.32 ± 0.83	0.19 ± 0.57	0.02 ± 0.26	-0.11 ± 0.64
			Synbiotic	0.54 ± 1.14	0.67 ± 1.52	0.73 ± 1.48*	0.13 ± 0.73	0.17 ± 0.80
	Selenomonadaceae	*Megamonas funiformis*	Control	0.73 ± 1.91	0.61 ± 1.47	0.61 ± 1.51	-0.12 ± 1.07	-0.12 ± 0.7
			Synbiotic	0.6 ± 1.77	0.6 ± 1.74	0.36 ± 1.15	-0.01 ± 1.7	-0.25 ± 1.66
	Veillonellaceae	*Megasphaera elsdenii*	Control	0.13 ± 0.29	0.15 ± 0.31	0.08 ± 0.18	0.02 ± 0.13	-0.05 ± 0.19
			Synbiotic	0.10 ± 0.34	0.09 ± 0.36	0.16 ± 0.53	-0.01 ± 0.39	0.11 ± 0.41*
		Veillonella ratti	Control	0.00 ± 0.00	0.00 ± 0.00	0.00 ± 0.00	0.00 ± 0.00	0.00 ± 0.00
			Synbiotic	0.26 ± 0.75*	0.49 ± 1.42*	0.39 ± 1.46	0.23 ± 0.70*	0.13 ± 1.05

Fusobacteriota	*Fusobacteriaceae*	*Fusobacterium mortiferum*	Control	0.93 ± 2.74	0.80 ± 2.62	1.45 ± 3.29	-0.13 ± 1.42	0.52 ± 2.31
			Synbiotic	0.54 ± 1.86	0.03 ± 0.11	0.19 ± 1.06*	-0.51 ± 1.76	-0.36 ± 1.19*
Verrucomicrobiota	*Akkermansiaceae*	*Akkermansia muciniphila*	Control	0.11 ± 0.40	0.10 ± 0.36	0.37 ± 1.51	-0.01 ± 0.51	0.26 ± 1.43
			Synbiotic	0.21 ± 0.68	0.06 ± 0.19	0.10 ± 0.38	-0.14 ± 0.64	-0.11 ± 0.79

Data are mean ± SD of relative abundance (%). * $p < 0.05$, ** $p < 0.01$ vs. Control. Each change is expressed as the value measured at 12 and 24 weeks minus the baseline value.

Table S4. Adverse events and changes in diabetes treatment.

	Control	Synbiotic
Adverse events		
Diarrhea	0	1
Flatulence	0	1
Soft stool	0	1
Vomiting	0	1
Changes in diabetes treatment		
New administration		
DPP-4 inhibitor	0	1
SGLT2 inhibitor	1	1
Glinide	1	0
Insulin	1	0
α-glucosidase inhibitor	0	0
Metformin	0	0
Thiazolidine	0	0
Discontinuation		
SU	1	0
Glinide	1	0
Dose up-titration		
Insulin	3	1
Metformin	0	0
Dose down-titration		
Insulin	1	0
Glinide	1	0
Metformin	0	0

SU, sulfonylurea; DPP-4 inhibitor, dipeptidyl peptidase-4 inhibitor; SGLT2 inhibitor, sodium-dependent glucose cotransporter-2 inhibitor.

References

1. Matsuda K, Tsuji H, Asahara T, Kado Y, Nomoto K. Sensitive quantitative detection of commensal bacteria by rRNA-targeted reverse transcription-PCR. Appl Environ Microbiol 2007;73: 32-39.
2. Matsuda K, Tsuji H, Asahara T, Matsumoto K, Takada T, Nomoto K. Establishment of an analytical system for the human fecal microbiota, based on reverse transcription-quantitative PCR targeting of multicopy rRNA molecules. Appl Environ Microbiol 2009;75: 1961-1969.
3. Sakaguchi S, Saito M, Tsuji H, Asahara T, Takata O, Fujimura J, et al. Bacterial rRNA-targeted reverse transcription-PCR used to identify pathogens responsible for fever with neutropenia. J Clin Microbiol 2010;48: 1624-1628.
4. Kikuchi E, Miyamoto Y, Narushima S, Itoh K. Design of species specific primers to identify 13 species of *Clostridium* harbored in human intestinal tracts. Microbiol Immunol 2002;46: 353–358.
5. Matsuki T, Watanabe K, Fujimoto J, Miyamoto Y, Takada T, Matsumoto K, et al. Development of 16S rRNA-gene-targeted group-specific primers for the detection and identification of predominant bacteria in human feces. Appl Environ Microbiol 2002;68: 5445-5451.
6. Matsuki T, Watanabe K, Fujimoto J, Takeda T, Tanaka R. Use of 16S rRNA gene-targeted group-specific primers for real-time PCR analysis of predominant bacteria in human feces. Appl Environ Microbiol 2004;70: 7220-7228.
7. Matsuki T. Development of quantitative PCR detection method with 16S rRNA gene-targeted genus- and species-specific primers for the analysis of human intestinal microflora and its application. Nihon Saikingaku Zasshi 2007;62: 255-261. [Article in Japanese]
8. Derrien M. Mucin utilisation and host interactions of the novel intestinal microbe *Akkermansia muciniphila*. Ph.D. thesis (ISBN 978-90-8504-644-8).
9. Wageningen University, Wageningen, The Netherlands, 2007.
10. Fujimoto J, Matsuki T, Sasamoto M, Tomii Y, Watanabe K. Identification and quantification of *Lactobacillus casei* strain Shirota in human feces with strain-specific primers derived from randomly amplified polymorphic DNA. Int J Food Microbiol 2008;126: 210-21
11. Fujimoto J, Tanigawa K, Kudo Y, Makino H, Watanabe K. Identification and quantification of viable Bifidobacterium breve strain Yakult in human faeces by using strain-specific primers and propidium monoazide. J Appl Microbiol 2010; 110:209-217.
